# Admixture in Humans of Two Divergent *Plasmodium knowlesi* Populations Associated with Different Macaque Host Species

**DOI:** 10.1371/journal.ppat.1004888

**Published:** 2015-05-28

**Authors:** Paul C. S. Divis, Balbir Singh, Fread Anderios, Shamilah Hisam, Asmad Matusop, Clemens H. Kocken, Samuel A. Assefa, Craig W. Duffy, David J. Conway

**Affiliations:** 1 Pathogen Molecular Biology Department, London School of Hygiene and Tropical Medicine, London, United Kingdom; 2 Malaria Research Centre, Faculty of Medicine and Health Sciences, University Malaysia Sarawak, Kuching, Sarawak, Malaysia; 3 Sabah State Public Health Laboratory, Kota Kinabalu, Sabah, Malaysia; 4 Institute for Medical Research, Kuala Lumpur, Malaysia; 5 Sarawak State Health Department, Kuching, Sarawak, Malaysia; 6 Biomedical Primate Research Centre, Rijswijk, The Netherlands; Arizona State University, UNITED STATES

## Abstract

Human malaria parasite species were originally acquired from other primate hosts and subsequently became endemic, then spread throughout large parts of the world. A major zoonosis is now occurring with *Plasmodium knowlesi* from macaques in Southeast Asia, with a recent acceleration in numbers of reported cases particularly in Malaysia. To investigate the parasite population genetics, we developed sensitive and species-specific microsatellite genotyping protocols and applied these to analysis of samples from 10 sites covering a range of >1,600 km within which most cases have occurred. Genotypic analyses of 599 *P*. *knowlesi* infections (552 in humans and 47 in wild macaques) at 10 highly polymorphic loci provide radical new insights on the emergence. Parasites from sympatric long-tailed macaques (*Macaca fascicularis*) and pig-tailed macaques (*M*. *nemestrina*) were very highly differentiated (FST = 0.22, and K-means clustering confirmed two host-associated subpopulations). Approximately two thirds of human *P*. *knowlesi* infections were of the long-tailed macaque type (Cluster 1), and one third were of the pig-tailed-macaque type (Cluster 2), with relative proportions varying across the different sites. Among the samples from humans, there was significant indication of genetic isolation by geographical distance overall and within Cluster 1 alone. Across the different sites, the level of multi-locus linkage disequilibrium correlated with the degree of local admixture of the two different clusters. The widespread occurrence of both types of *P*. *knowlesi* in humans enhances the potential for parasite adaptation in this zoonotic system.

## Author Summary

Extraordinary phases of pathogen evolution may occur during an emerging zoonosis, potentially involving adaptation to human hosts, with changes in patterns of virulence and transmission. In a large population genetic survey, we show that the malaria parasite *Plasmodium knowlesi* in humans is an admixture of two highly divergent parasite populations, each associated with different forest-dwelling macaque reservoir host species. Most of the transmission and sexual reproduction occurs separately in each of the two parasite populations. In addition to the reservoir host-associated parasite population structure, there was also significant genetic differentiation that correlated with geographical distance. Although both *P*. *knowlesi* types co-exist in the same areas, the divergence between them is similar to or greater than that seen between sub-species in other sexually reproducing eukaryotes. This may offer particular opportunities for evolution of virulence and host-specificity, not seen with other malaria parasites, so studies of ongoing adaptation and interventions to reduce transmission are urgent priorities.

## Introduction

The epidemiological emergence of infections can be traced by genotypic analyses, with a high level of resolution when pathogens have a high mutation rate, as illustrated by recently emerged viruses that now have a massive impact on global public health [1,2]. Such analysis is more challenging for eukaryote pathogens with low mutation rate, although it is now clear that the major human malaria parasites *Plasmodium falciparum* and *P*. *vivax* have been endemic for many thousands of years after having been acquired as zoonotic infections from African apes [3,4]. In contrast, natural human infections by *P*. *knowlesi* were almost unknown [5] until a large focus of cases in Malaysian Borneo was described a decade ago [6]. Infections have since been reported from throughout southeast Asia, within the geographical range of the long-tailed and pig-tailed macaque reservoir hosts (*Macaca fascicularis* and *M*. *nemestrina*) and mosquito vectors (of the *Anopheles leucosphyrus* group) [7]. The most highly affected country is Malaysia, where there have been thousands of reported cases and *P*. *knowlesi* is now the leading cause of malaria in most areas [8,9].

It is vital to determine the causes of this apparent emergence, as *P*. *knowlesi* can cause severe clinical malaria with a potentially fatal outcome [10–12]. Increasing rates of case detection may reflect better diagnosis, increased transmission by mosquitoes from reservoir host macaques to humans, or parasite adaptation to humans. Molecular tools to discriminate *P*. *knowlesi* from other malaria parasite species were not widely applied until the zoonosis became known, but analysis of DNA in archived blood samples from Malaysia and Thailand shows that it was already widespread twenty years ago [13,14]. Sequences of parasite mitochondrial genomes and a few nuclear gene loci indicate ongoing zoonotic infection, as human *P*. *knowlesi* genotypes share most alleles identified in parasites sampled from wild macaques [15–17].

To understand this zoonosis, and to identify whether human-to-human mosquito transmission is occurring, analyses of parasite population genetic structure in humans and macaques should be performed by extensive population sampling and characterisation of multiple putatively neutral loci. This study presents a *P*. *knowlesi* microsatellite genotyping toolkit and its application to the analysis of a large sample of isolates from human cases at ten different sites, as well as from both species of wild macaque reservoir hosts. Results reveal a profound host-associated sympatric subdivision within this parasite species, as well as geographical differentiation indicating genetic isolation by distance. The existence of two divergent parasite subpopulations, and their admixture in human infections provides unparalleled opportunity for parasite hybridisation and adaptation. Observations of some clinical infections with parasite types that appear intermediate between the two subpopulations may reflect this process, and are a possible result of human-to-human mosquito transmission.

## Results

### *P*. *knowlesi* microsatellites as genetic markers for population studies

Hemi-nested PCR assays were developed for amplification of 19 tri-nucleotide simple sequence repeat loci from throughout the genome of *P*. *knowlesi* and tested for species-specificity using control DNA from all 10 known parasite species of humans, long-tailed or pig-tailed macaques, as well as human and macaque DNA to identify those suitable for genotyping samples from all hosts (S1 Table). Assays for 11 loci were entirely species-specific for *P*. *knowlesi*, and 10 of these gave a clear single electrophoretic peak for each allele without any stutter bands (S2 Table). These were used to genotype *P*. *knowlesi* infections in a total of 599 humans and wild macaques with a high rate of success, 556 (92.8%) scoring clearly for all 10 loci (S3 Table and S1 Dataset). Numbers of alleles at each locus ranged from 7 (for locus NC03_2) to 21 (for locus CD05_06) (S1 Dataset).

### Host-dependent genetic structure of *P*. *knowlesi*

**Fig 1:**
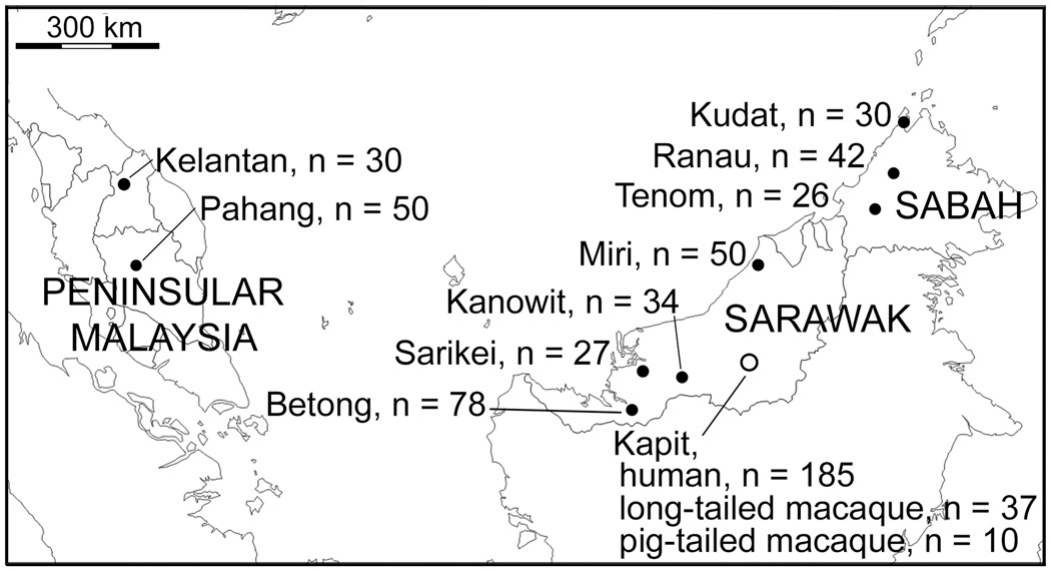
Map of sampling locations of 599 *P*. *knowlesi* infections genotyped in this study. A total of 552 samples were from *P*. *knowlesi* malaria patients from 10 geographical locations: Peninsular Malaysia (Kelantan and Pahang), Sarawak (Kapit, Betong, Miri, Kanowit and Sarikei), and Sabah (Kudat, Ranau and Tenom). Additionally, 47 samples were from wild macaques (37 long-tailed and 10 pig-tailed macaques) with *P*. *knowlesi* infections in Kapit.

**Fig 2:**
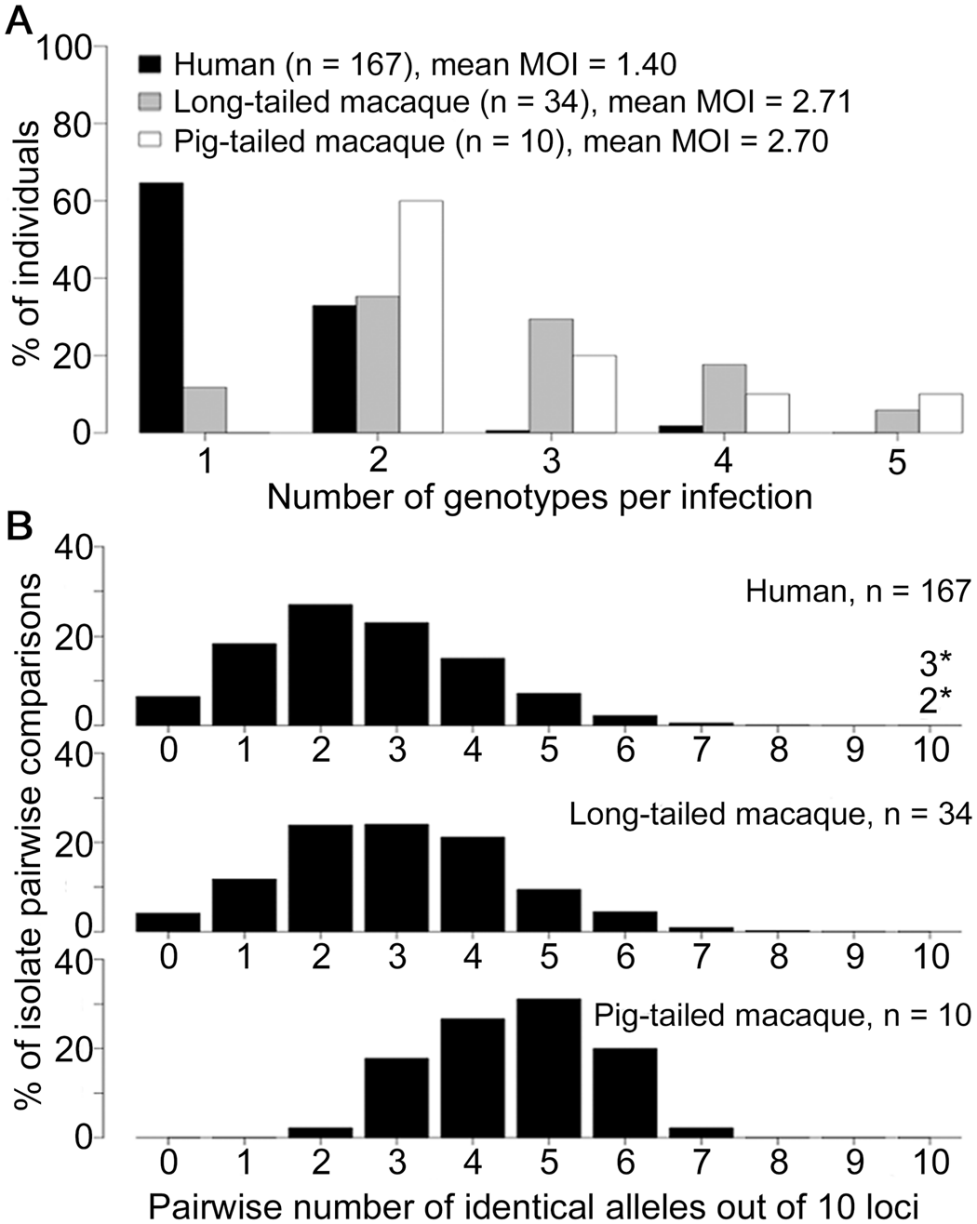
Multiple genotype *P*. *knowlesi* infections and diversity among infections in three host species from Kapit. All data for the multiple genotype infections and distribution of identical alleles between infections derived from complete genotyping of 10 microsatellite loci. (A) Numbers of different *P*. *knowlesi* genotypes per infection (multiplicity of infection, MOI) showing significant difference between human and macaque infections (Fisher’s Exact P < 1 x 10^–15^), but not between long-tailed and pig-tailed macaques (Fisher’s Exact P = 0.65). (B) Numbers of identical alleles out of 10 loci in pairwise comparisons of infections, showing a similar diversity among infections in humans and long-tailed macaques, but a higher average identity among infections from pig-tailed macaques. All infections had a different 10-locus genotype, except for 5 of the 167 human infections (there was one pair sharing an identical 10-locus genotype, and a triplet of infections sharing another 10-locus genotype, indicated with asterisks here and shown in S4 Table).

We first compared parasites from different host species sampled from Kapit where high numbers of clinical cases are seen (Fig 1), analysing the infections with complete 10-locus genotype data. Almost all *P*. *knowlesi* infections in macaques contained multiple genotypes, with no significant difference between long-tailed macaques (88% of 34 were mixed, a mean of 2.71 genotypes per infection) and pig-tailed macaques (100% of 10 were mixed, a mean of 2.70 genotypes per infection; P = 0.65 for comparison between macaque host species), whereas only a minority of human *P*. *knowlesi* infections had multiple genotypes (35% of 167, a mean of 1.40 genotypes per infection; P < 10^–15^ for comparison between humans and macaques) (Fig 2A). To allow equally weighted sampling per host, the predominant allele at each locus within each infection was counted for subsequent analysis (S1 Dataset).

Pairwise comparisons of each of the complete 10-locus profiles revealed that all infections in Kapit were genotypically distinct, except for one identical pair and one identical triplet of human infections (Fig 2B, S4 Table). There was a much higher average proportion of shared alleles among pig-tailed macaque infections than among those in long-tailed macaques or humans (medians of 5, 3 and 2 identical alleles out of 10 loci respectively). Analysis of allele frequencies revealed that *P*. *knowlesi* parasites from pig-tailed macaques are very highly divergent from those in long-tailed macaques (*F*_*ST*_ = 0.217, P < 0.001), whereas those in humans have an intermediate level of relatedness (*F*_*ST*_ = 0.067 versus long-tailed macaques, *F*_*ST*_ = 0.104 versus pig-tailed macaques; P < 0.001 for both).

**Fig 3:**
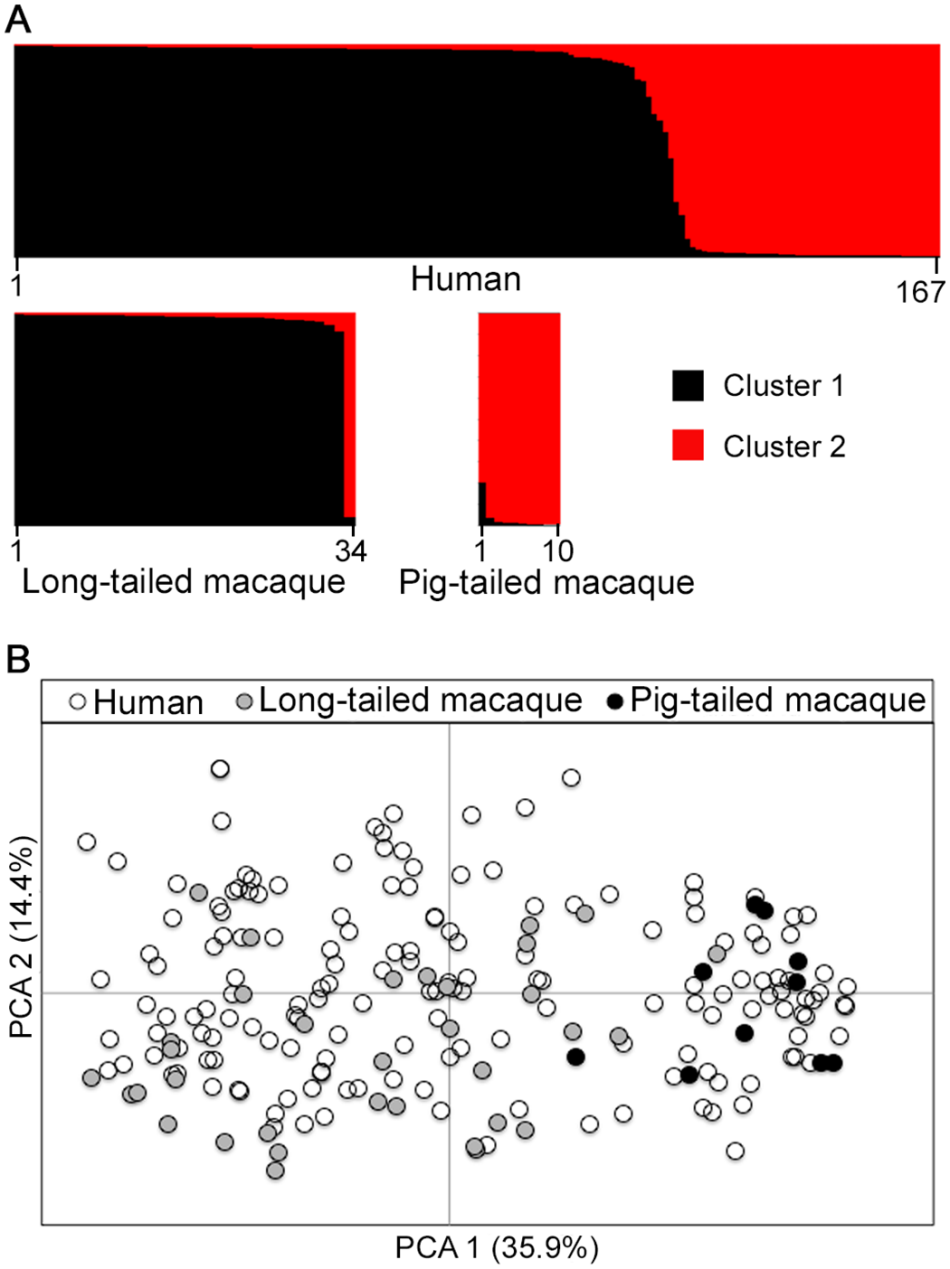
Population genetic structure of *P*. *knowlesi* from infections in three host species in Kapit. Both STRUCTURE and principal component analyses were conducted based on the complete 10-locus genotype dataset (167 from humans, 34 from long-tailed macaques, 10 from pig-tailed macaques). (A) Bayesian model-based STRUCTURE analysis indicates two subpopulation clusters throughout the whole dataset (*K* = 2, *ΔK* = 936.75), with almost complete partitioning between the two macaque host species. Cluster 1 is shown in black while Cluster 2 is in red. (B) Principal component analysis (PCA) of the genetic divergence among all infections. The percentage of variation captured by each of the first two principal components is shown in brackets. Infections from the different macaque host species are almost completely separated by the first principal component, while human infections are distributed throughout the full range on both axes.

A Bayesian model-based STRUCTURE analysis of multi-locus genotype data from all hosts sampled in Kapit clearly indicated the existence of two sub-population clusters of *P*. *knowlesi* (*K* = 2; *ΔK* = 936.75 based on Evanno’s estimation of *K*-population) (Fig 3A, S1 Fig and S1 Dataset). An individual infection genotype was assigned to be predominantly of a particular cluster if the STRUCTURE analysis score exceeded 0.5 for that cluster. All except one of the long-tailed macaque infections were assigned to the Cluster 1 subpopulation, whereas all pig-tailed macaque infections were assigned to the Cluster 2 subpopulation, while 71% of human infections were assigned to Cluster 1 and 29% to Cluster 2 (Fig 3A, S5 Table). A small minority of those which were primarily assigned to either cluster appeared to have a degree of mixed assignment, with scores nearer 0.5 than either zero or 1.0 for the alternative clusters (S1 Dataset), which is analysed in a separate section below. An independent scan by principal component analysis (PCA) showed an almost complete separation between parasites from long-tailed macaques and pig-tailed macaques along the first principal component, while parasites from humans covered the whole distribution and overlapped with all of the samples from both of the macaque hosts (Fig 3B).

### Geographical population genetic structure of *P*. *knowlesi*

**Fig 4:**
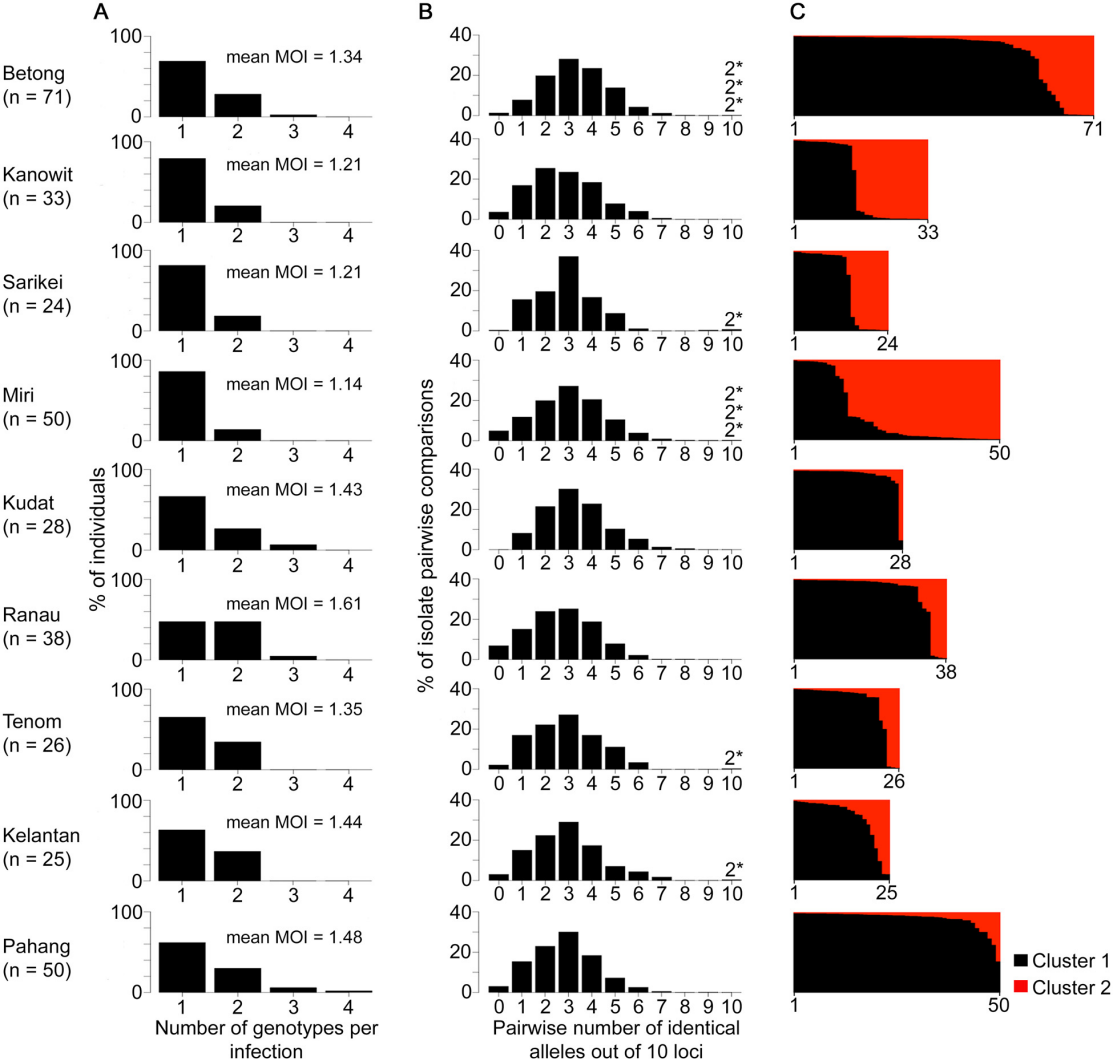
Diversity and genetic structure of *P*. *knowlesi* in human infections from nine different geographical locations. All *P*. *knowlesi* data for analysing multiple genotype infections, distribution of identical alleles between isolates and STRUCTURE analysis were derived from complete genotyping of 10 microsatellite loci. (A) Proportions of infections containing different numbers of genotypes (multiplicity of infection, MOI). Comparison across all 10 geographical locations including Kapit (data in Fig 2) showed no significant differences (Pearson’s *X* ^*2*^ with 10000 replicates, P = 0.096). (B) Distribution of numbers of identical alleles out of 10 loci in pairwise comparisons of infections. In six of the populations, a small number of pairs of identical multi-locus genotypes were seen as indicated with a label here (2*) and tabulated in detail in S4 Table. (C) Subpopulation clusters inferred by the Bayesian model-based STRUCTURE analysis with Cluster 1 (black) and Cluster 2 (red) corresponding to those identified in Fig 3 (*K* = 2, *ΔK* = 174.94). Proportions of isolates assigned as Cluster 2 are highest at the sites in Sarawak (top four panels in the figure; locations of all sites are shown in Fig 1).

We analysed a further 367 human *P*. *knowlesi* infections from nine other geographical sites (Fig 1). Most human infections had single *P*. *knowlesi* genotypes (Fig 4A and S1 Dataset), and there were no differences in the proportions of mixed genotype infections across all sites (Comparison across 10 sites including Kapit: Pearson’s *X* ^*2*^, P = 0.096; 32% of infections having > 1 genotype overall). There were no differences in allelic diversity among the different sites (*H*_*E*_ estimates between 0.67 and 0.75, P > 0.1 for all pairwise Wilcoxon Signed Rank tests across all 10 loci, S3 Table). Pairwise comparisons among genotypes from different infections showed a similar level of diversity at each site, with a median of 2 or 3 identical alleles out of 10 loci in each site (Fig 4B, S4 Table). Every infection had a different multi-locus genotype, and there were virtually none that shared alleles at more than 7 loci, except for nine pairs of identical haplotypes (three pairs in Betong, three in Miri, and one in each of Sarikei, Tenom and Kelantan) (Fig 4B). Each identical haplotype pair was shared by infections from different individuals sampled at the same site within the same year, except for two of the identical haplotype pairs in Miri, shared by individual infections sampled one and two years apart (S4 Table).

**Fig 5:**
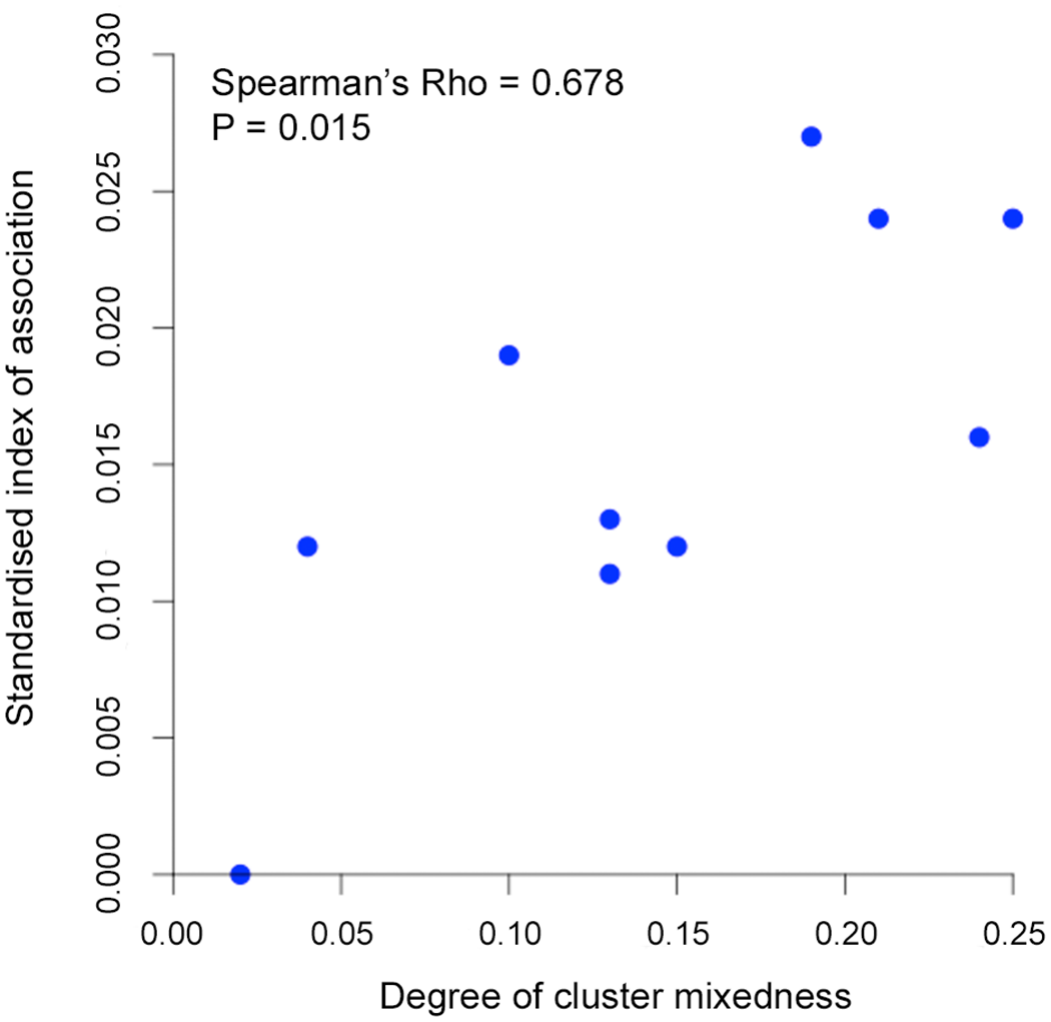
Correlation between degree of cluster admixture and multi-locus linkage disequilibrium (standardised index of association). The degree of *P*. *knowlesi* cluster admixture was estimated as the local cluster mixedness (p1*p2) based on the proportions of infections designated as Cluster 1 (*p1*) and Cluster 2 (*p2*) at each of 10 sampling sites for human infections across Malaysia (Spearman’s Rho = 0.678, P = 0.015). S5 Table gives details of each of these values for each population.

There were two subpopulation clusters (*K* = 2, *ΔK* = 174.94, S1 Fig) throughout all of these sites, as had been seen in Kapit, but the relative frequency of the clusters varied geographically (P < 0.0001, Fig 4C). The Cluster 1 subpopulation was more frequent overall, but Cluster 2 was also common at each of the sites in Sarawak, particularly in Miri and Kanowit where it was more frequent than Cluster 1 (S5 Table and S1 Dataset). Over all human infections, there was a similarly high level of divergence in allele frequencies between the two subpopulation clusters as was seen between parasites from the two different macaque host species (*F*_*ST*_ = 0.194, P < 0.001). As expected, the degree of cluster admixture at each sampling site (p1*p2, where *p1* and *p2* are the local frequencies of Cluster 1 and Cluster 2 respectively) correlated positively with the (*I* ^S^_A_) index of multi-locus linkage disequilibrium (Spearman’s Rho = 0.678, P = 0.015, Fig 5 and S5 Table).

**Fig 6:**
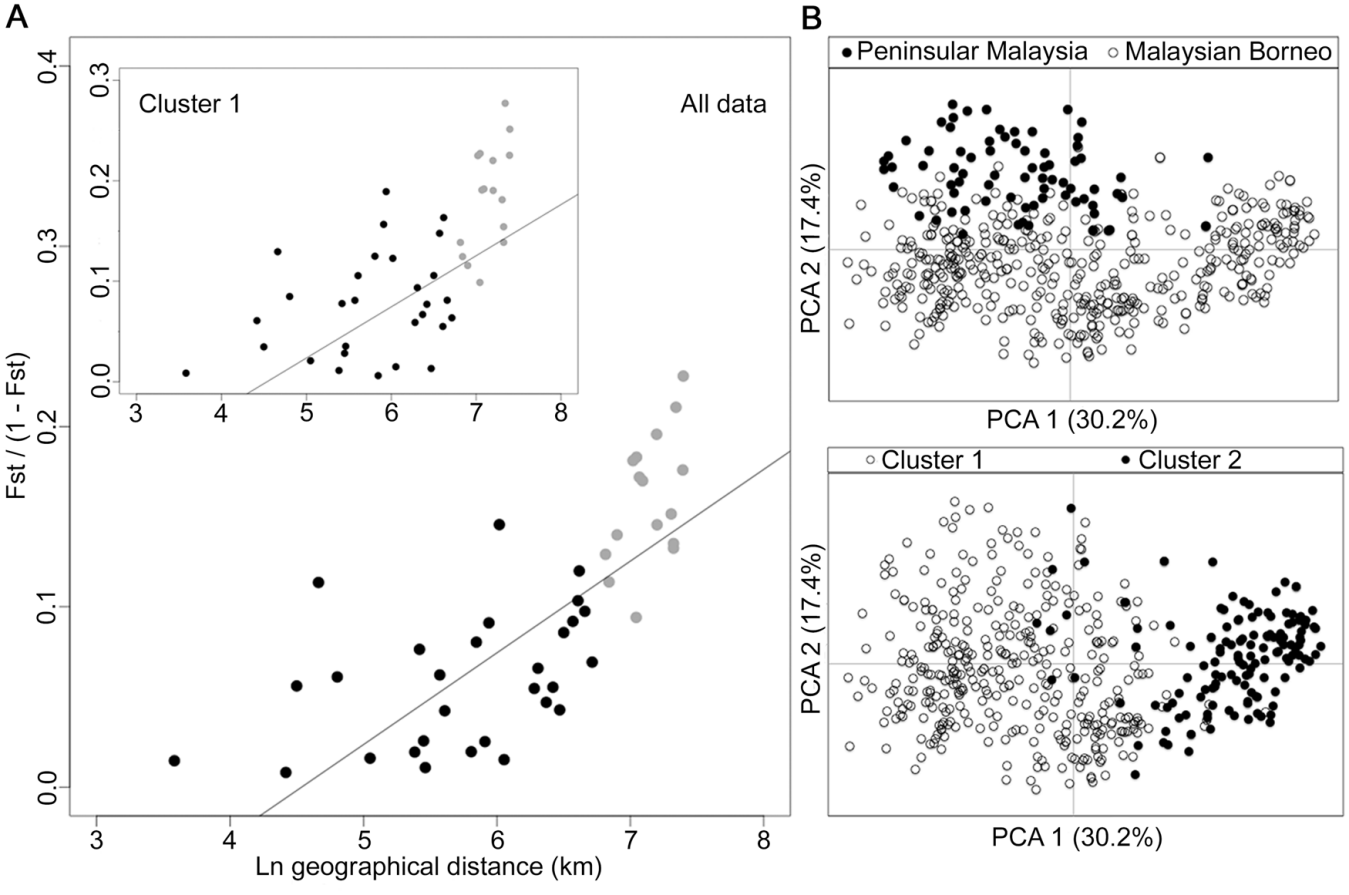
Isolation-by-distance model and principal component analysis (PCA) of the human *P*. *knowlesi* isolates. (A) Relationship between transformed genetic differentiation and natural log of geographical distance (Euclidean distances of population pairs ranged from 36 km to 1631 km) for all pairs of sites across Malaysia (Mantel test of matrix correlation P < 0.0001), with a similar relationship when analysing only isolates from the Cluster 1 subpopulation (Mantel test P = 0.0016). Black dots denote pairs of populations within Malaysian Borneo and Peninsular Malaysia, while grey dots denote pairs of populations between Malaysian Borneo and Peninsular Malaysia (all data are given in S6 Table). Evidence of isolation by distance remained when only sites within Borneo were considered (Mantel test P = 0.0016). (B) PCA of the whole infection haplotype dataset indicated differentiation of Peninsular Malaysia isolates from Malaysian Borneo isolates by the second principal component axis, whereas isolates defined as Cluster 1 and Cluster 2 by STRUCTURE analysis were almost completely differentiated along the first principal component axis.

Analysis of geographical divergence on the basis of *F*_ST_ indices derived from population allele frequencies (S6 Table) identified a pattern strongly consistent with isolation by distance (Mantel test of matrix correlation P < 0.0001, Fig 6A). The greatest level of divergence was seen between peninsular Malaysia and Borneo as expected, although isolation by distance was also apparent within Borneo (Mantel test P = 0.0016). The overall pattern consistent with isolation by distance remained when only infections with Cluster 1 genotypes were analysed (P = 0.0016, Fig 6A). There was a similar trend for the smaller number of samples with Cluster 2 genotypes, although this was not significant (P = 0.0922, S2 Fig), indicating that the majority of the geographical differentiation is independent of the Cluster subpopulation structure. A principal component analysis of all individual infection genotypes showed that most of the overall diversity is among those defined as Cluster 1 by the STRUCTURE analysis (Cluster 2 infections covered only part of the first principal component distribution), and infections from peninsular Malaysia are restricted to part of the second principal component distribution (Fig 6B).

Combination of the macaque samples together with all of the human samples across the 10 geographical locations confirmed the definition of the two *P*. *knowlesi* subpopulation clusters, which correspond to those shown above (S3 Fig). Allele frequency distributions showed that some loci were particularly differentiated between the subpopulation clusters, with *F*_*ST*_ > 0.3 for loci NC03_2 and CD13_61 (S4 Fig). The robustness of the two assigned clusters was confirmed even with the exclusion of these most highly differentiated loci in the STRUCTURE analysis (S5 Fig).

### Evaluation of cluster assignment indices

**Fig 7:**
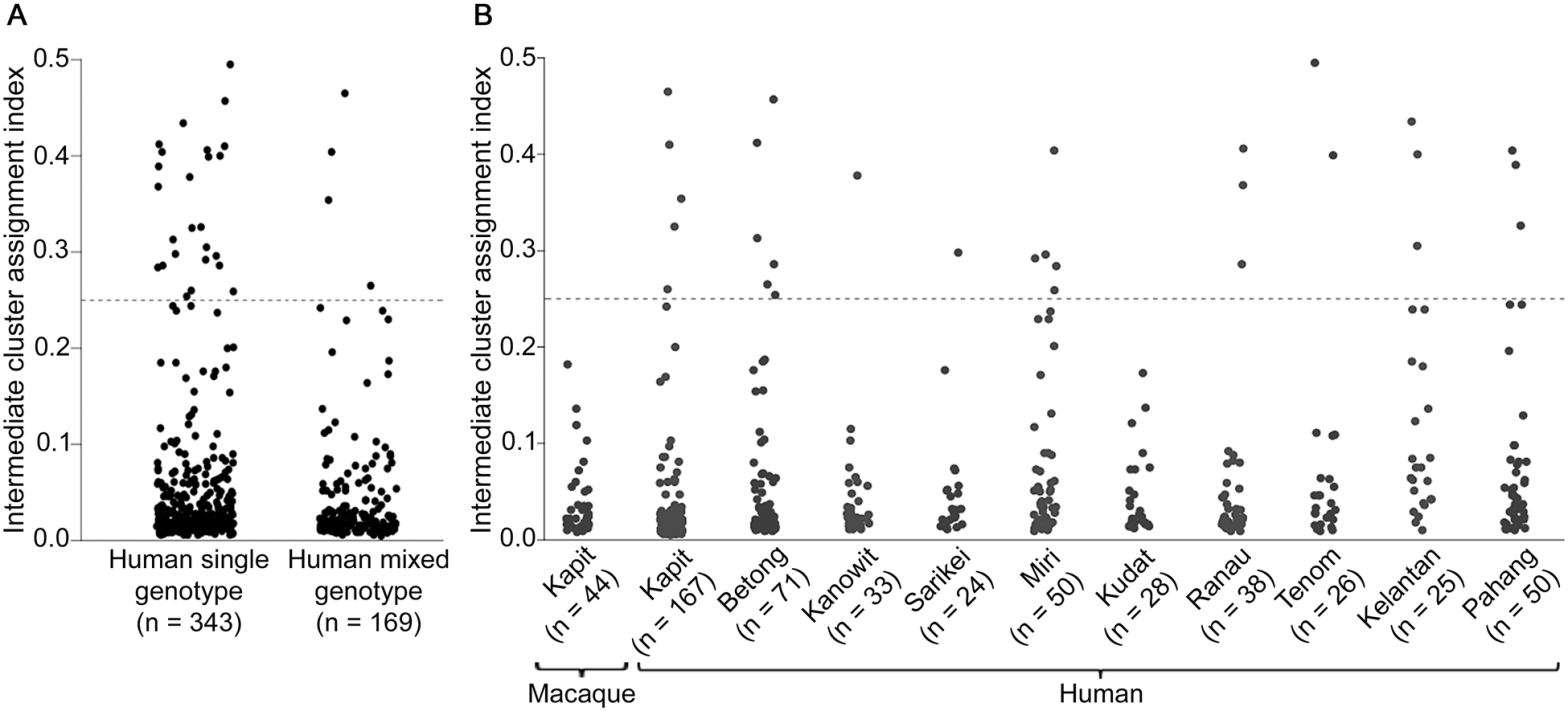
Intermediate cluster assignment indices in *P*. *knowlesi* infections in humans and macaques. The index for each infection was based on the proportion of shared ancestry between Cluster 1 and Cluster 2 inferred by the STRUCTURE analysis on all samples genotyped at 10 microsatellite loci. An index below 0.25 signifies the individual parasites were predominantly either Cluster 1 or Cluster 2 while those above 0.25 had more intermediate assignment (up to a maximum index value of 0.5). (A) There was no significant difference between single or multiple genotype human infections in the distribution of the indices (Mann-Whitney U test, P = 0.20). (B) Intermediate cluster assignment indices were lower in infections from macaques in Kapit than in human infections overall (Mann-Whitney U test, P < 0.001), but were not significantly different from human infections in Kapit (P = 0.25). Among geographical sites, the indices for human infections were significantly higher in Kelantan than in Kapit, Betong, Kanowit, Kudat or Ranau (P < 0.05 for each pairwise comparison after Bonferroni correction).

Most individual infection genotypes had a clear majority of putative ancestry assignment to either Cluster 1 or Cluster 2, but a small minority of infections had a more intermediate profile (Figs 3A and 4C). Quantitative analysis of the proportional Cluster 1 and Cluster 2 ancestry assignments for each infection genotype based on the STRUCTURE analysis yielded an index of the degree of intermediate cluster assignment for each infection. This has a maximum possible value of 0.5, although most infections had values closer to zero. The intermediate cluster assignment indices showed no difference between single and mixed genotype human infections (Mann-Whitney test P = 0.20, Fig 7A), whereas both of these independently had higher indices than the macaque infections (P < 0.001 for both comparisons). When analysis was focused on Kapit alone, the distribution of intermediate cluster assignment indices were not significantly different between human and macaque infections (P = 0.25, Fig 7B). However, there were geographical differences, with human infections from Kelantan having a significantly higher distribution of values compared to five of the sites in Borneo (Mann-Whitney test P < 0.05 for each comparison after Bonferroni correction, Fig 7B). Across the different sites, there was no significant correlation between the local population admixture of both clusters (p1*p2, S5 Table) and the mean or variance of intermediate cluster assignment indices (P = 0.33 and P = 0.59 respectively). Infections which had intermediate cluster assignment (index values > 0.25) were not particularly closely related, having a similar degree of allele sharing as seen in the general local populations (S6 Fig, compared with Figs 2B and 4B).

## Discussion

We show that human *P*. *knowlesi* is an admixture of two divergent parasite populations associated with different forest-dwelling macaque reservoir hosts. In human infections, the long-tailed macaque-associated *P*. *knowlesi* type (Cluster 1) is most common overall and at most of the geographical sites, while the pig-tailed macaque-associated type (Cluster 2) is also common at sites in Sarawak. The estimate of divergence between these two sympatric parasite subpopulations (*F*_ST_ index of ~ 0.22 averaged over 10 microsatellite loci) may be conservative, due to high allelic diversity of the microsatellite loci which restricts the potential upper range of fixation indices [18,19]. The differentiation varied among the loci, with two of the microsatellite loci being particularly highly differentiated between the clusters (*F*_*ST*_ ~ 0.35), so the robustness of the two assigned clusters was confirmed by repeat analyses which excluded these. Previous analysis of *P*. *knowlesi* mitochondrial DNA sequences from a relatively small number of human and long-tailed macaque infections in Kapit did not indicate two divergent lineages [15], although analysis of samples from Sabah suggests that sequences from pig-tailed macaque infections are differentiated from sequences from long-tailed macaque infections [20].

The results confirm that humans have mostly single genotype *P*. *knowlesi* infections whereas macaques have polyclonal infections, supporting the expectation that there is a higher rate of transmission among macaques [15,21]. The estimated number of genotypes per infection here is a minimum number based on the alleles detected, and it is possible that some infections may have contained additional parasite clones that were not detected, due to having low density in the blood or having similar alleles to the ones detected. The number of *P*. *knowlesi* genotypes detected per infection in humans is lower than was previously seen in microsatellite analyses of the endemic human malaria parasites *P*. *falciparum* and *P*. *vivax* in some of the same areas in Malaysia [22,23], whereas the number of *P*. *knowlesi* genotypes per infection in macaques is much higher. Levels of multi-locus linkage disequilibrium in *P*. *knowlesi* here are lower than reported in *P*. *vivax* or *P*. *falciparum* in these areas [22,23], indicating that recombination in *P*. *knowlesi* probably commonly occurs in mosquitoes containing a macaque blood meal with multiple parasite genotypes.

It is unknown how the two sympatric *P*. *knowlesi* subpopulations are genetically isolated. The observation of a single long-tailed macaque with a *P*. *knowlesi* Cluster 2 type infection (otherwise only seen in pig-tailed macaques and humans) suggests there is not an absolute barrier in terms of primate host susceptibility, although there are differences in ecology. Additional sampling of both long-tailed and pig-tailed macaques will be important to confirm the host associations of different parasites [20]. Both macaque species are widespread, but long-tailed macaques prefer secondary forest near human settlements where they have access to farms for food, whereas pig-tailed macaques spend more time in ground foraging in primary forests, generally having less frequent contact with humans [24]. There may be differential susceptibility of mosquito species to the respective parasite types, as suggested for subpopulations of another malaria parasite elsewhere [25], or different mosquitoes may feed on the respective macaque host species. Genetic differentiation in *P*. *knowlesi* was also strongly correlated with geographical distance, overall and for the Cluster 1 parasites. The observation of highest *F*_*ST*_ values between populations from Malaysian Borneo and Peninsular Malaysia was expected, as the South China Sea has separated macaques in these areas since the last glacial period [26], but a test for isolation by distance remained significant when analysing only sites within Borneo.

A small minority of human infections had intermediate cluster assignment indices, which could potentially result from occasional crossbreeding between the two genotypic clusters, although this cannot be concluded from these data alone. Hybridisation between species or sub-species can offer opportunities for adaptation, and has been associated with emergence of novel host-specificity or pathogenicity in other parasitic protozoa [27] and fungi [28]. Switching of host species has occurred repeatedly in malaria parasites of birds [29] and small mammals [30], as well as apes and humans [3,4], but the occurrence of parasite hybridisation and introgression has not been investigated. The potential occurrence of inter-cluster hybridisation in even a minority of human *P*. *knowlesi* infections, combined with the possibility of human-mosquito-human transmission, may increase the potential for *P*. *knowlesi* adaptation to the human host or to mosquito species that are more abundant than the currently known forest-associated vectors.

Genome-wide analysis of *P*. *knowlesi* populations would enable further evaluation of the genetic structure of this zoonotic parasite species, and allow scanning for loci under selection within each of the two subpopulations. Human clinical isolates containing single species infections would be relatively straightforward to analyse, as *P*. *knowlesi* sequences would be unmixed with those of other human malaria species. In contrast, as natural macaque infections usually contain a mixture of different malaria parasite species [15], to obtain unambiguous genome sequences it may be necessary to sequence from individual parasites isolated from these hosts [31]. Although experimental studies on *P*. *knowlesi* are usually conducted *in vivo* in non-human primates [32–34], new approaches to adapt the parasites to *in vitro* growth using human erythrocytes have been successful [35,36]. Analysis of phenotypic differences between the different host-associated types may be investigated using both *in vivo* and *in vitro* experimental systems, while continued epidemiological and clinical surveillance for increasing incidence or disease severity is of the highest priority.

## Materials and Methods

### Ethics statement

Human blood samples were taken after written informed consent had been obtained from patients. This study was approved by the Medical Research and Ethics Committee of the Malaysian Ministry of Health (Reference number: NMRR-12-1086-13607), which operates in accordance to the International Conference of Harmonization Good Clinical Practice Guidelines. Animal sampling was carried out as previously described [15] in strict accordance with the recommendations by the Sarawak Forestry Department for the capture, use and release of wild macaques. A veterinarian took a venous blood sample from each macaque following anesthesia by intramuscular injection of tiletamine and zolazepam, and all efforts were made to minimize suffering by collecting blood at the trap sites and releasing the animals immediately after the blood samples had been obtained. The Sarawak Forestry Department approved the study protocol for capture, collection of blood samples and release of wild macaques (Permit Numbers: NPW.907.4.2–32, NPW.907.4.2–97, NPW.907.4.2–98, 57/2006 and 70/2007). A permit to access and collect macaque blood samples for the purpose of research was also obtained from the Sarawak Biodiversity Centre (Permit Number: SBC-RP-0081-BS).

### *P*. *knowlesi* samples from humans and macaques

A total of 599 DNA samples from different *P*. *knowlesi* infections of humans and macaques were analysed from collections performed at 10 different geographical sites (Fig 1). 552 samples were from human *P*. *knowlesi* malaria patients from all of the sites, eight in Malaysian Borneo (Sarawak and Sabah states) and two in Peninsular Malaysia (Kelantan and Pahang states). For samples from Sarawak, DNA was extracted at the University Malaysia Sarawak (UNIMAS) in Kuching from previously reported blood samples collected between 2000 and 2011 [6,10,37,38] as well as new samples collected in 2012 and 2013, allowing analysis of five sites: Kapit (n = 185), Betong (n = 78), Kanowit (n = 34), Sarikei (n = 27) and Miri (n = 50). Samples from Sabah were collected in 2013, and DNA was extracted by the Sabah Public Health Reference Laboratory, allowing analysis of three sites: Kudat (n = 30), Ranau (n = 42) and Tenom (n = 26). For Peninsular Malaysia, blood samples collected from Kelantan (n = 30) and Pahang (n = 50) underwent DNA extraction at the Institute for Medical Research in Kuala Lumpur. We analysed DNA from blood samples of a total of 47 wild macaques (long-tailed macaque, *Macaca fascicularis* n = 37; pig-tailed macaque, *M*. *nemestrina* n = 10) previously collected within 30 km radius of Kapit town in Sarawak [15]. The map locations and dates of the individual macaque sampling is shown in S7 Fig. The presence of *P*. *knowlesi* DNA was confirmed in all samples at UNIMAS by nested PCR assays [6,15].

### Development of *P*. *knowlesi* microsatellite genotyping markers

A combination of three microsatellite mining tools (iMEX [39], mreps [40], and MSATCOMMANDER [41]) were used to identify simple sequence repeat loci from the *P*. *knowlesi* reference genome [42]. Loci with perfect tri-nucleotide simple repeat sequences were carefully selected using customised perl-script commands based on narrow criteria to maximise their likely utility for genotyping: i) a minimum of 7 repeat copies in each microsatellite in the reference sequence, ii) located at non-telomeric chromosomal regions as defined by regions syntenic with the *P*. *vivax* reference genome [43], iii) absence of any homopolymeric tracts adjacent to the microsatellite sequence that could give rise to additional size polymorphism. As a result, 19 trinucleotide repeat loci widely spaced in the genome (S1 Table) were shortlisted and PCR primers were designed using PrimerSelect software (DNASTAR, USA) for hemi-nested PCR assays. The specificity of PCR was tested using DNA controls of all human *Plasmodium* species, common malaria parasites of the Southeast Asian macaques (*P*. *knowlesi*, *P*. *coatneyi*, *P*. *inui*, *P*. *cynomolgi* and *P*. *fieldi*), as well as human, long-tailed and pig-tailed macaque DNA. Loci for which primers showed complete specificity of amplification from *P*. *knowlesi* were tested further for genotyping performance.

### PCR and genotyping protocols

Genotyping of each microsatellite locus was performed using a hemi-nested protocol with a fluorescent dye-labelled inner primer during the second round PCR amplification (primers listed in S1 Table). Both first and second round PCR amplifications were conducted in individual tubes or wells for each locus, in 11 μl reaction volume containing 0.2 mM each dNTP (Bioline, UK), 2 mM MgSO_4_, 1X ThermoPol II reaction buffer (NEB, UK), 0.275 U *Taq* DNA polymerase (NEB, UK), 0.1 μM of each forward and reverse primer, and 1 μl sample DNA template. The PCR cycling conditions were as follows: initial denaturation at 94°C for 2 min, followed by 28 cycles of 94°C for 30 sec, annealing at 56°C for 30 sec and elongation at 68°C for 30 sec, with a final elongation step at 68°C for 1 min. Final PCR products were pooled into three groups of loci with different product size and dye profiles together with Genescan 500 LIZ molecular size standards (Applied Biosystems, UK) and run on a Genetic Analyzer 3730 capillary electrophoretic system (Applied Biosystems, UK). GENEMAPPER version 4.0 software (Applied Biosystems, UK) was used for scoring of allele electrophoretic size, and quantification of peak heights.

### Genotypic and statistical analyses

Infections containing multiple haploid parasite genotypes were apparent as multiple electrophoretic peaks for a locus corresponding to different alleles. The apparent genotypic multiplicity of infection (MOI) was determined by the locus with the most alleles detected in the infection, considering peaks with height of at least 25% relative to the predominant allele within each isolate. The predominant allele per locus within each infection was counted for subsequent population genetic analyses. Allelic diversity at each locus was measured as the virtual heterozygosity (*H*_*E*_) using FSTAT software version 2.9.3.2 (http://www2.unil.ch/popgen/softwares/fstat.htm), and allele frequency distributions were also inspected using GenAlEx version 6 [44] within the Microsoft Excel platform. Genetic differentiation between each population was measured by pairwise fixation indices (*F*_*ST*_) using FSTAT, with Bonferroni correction on a nominal significance level of 0.05 applied for multiple comparisons across the population pairs. To test for correlation between genetic differentiation and geographical distance, a Mantel test for isolation by distance was performed with Rousset’s linearised *F*_*ST*_/(1-*F*_*ST*_) plotted against the natural log of geographic distance using Genepop version 4.2 [45].

The relatedness of haplotypes between individual isolates was assessed by measuring the pairwise proportion of shared alleles, excluding samples with missing data at any locus. A matrix of pairwise similarity among isolates was calculated based on the identical or mismatched alleles from a complete set of loci and the distribution of shared alleles between sample pairs for each population was visualised using a customised perl-script command. To test for non-random allele assortment, multi-locus linkage disequilibrium (LD) was assessed by the standardised index of association (*I*_*A*_^*S*^) using LIAN version 3.6 [46], with significance of the *I*_*A*_^*S*^ values tested by Monte-Carlo simulation with 10,000 data permutations to generate the null distribution under linkage equilibrium.

To explore evidence of population substructure in the entire population, a Bayesian analysis was performed using the STRUCTURE version 2.3.4 software [47] using samples with no missing data at any locus. Individuals in the population pool were clustered to the most likely population (*K*) by measuring the probability of ancestry using the multi-locus genotype data. The program parameters were set to admixture model with correlated allele frequency, with 50,000 burn-in period and 100,000 Markov chain (MCMC) iterations. To run the simulation, *K* value was predefined from 1–10 and the run was performed in 20 replicates for each *K*. The most probable *K* value was then calculated according to Evanno’s method [48] using the webpage interface STRUCTURE Harvester [49]. The assignment of a sample to a subpopulation cluster was based on the inferred cluster scores by STRUCTURE analysis, where samples with inferred cluster scores within a range in relation to the *K*-value were assigned together as one subpopulation cluster. The intermediate cluster assignment indices were calculated based on the proportion of shared cluster ancestries per individual isolate inferred by the cluster scores from the STRUCTURE analysis.

We also independently performed a principal component analysis (PCA) using the GenAlEx package for the same purpose. Samples with missing data at any locus were excluded, and the genetic distance matrix was generated based on the allelic mismatches between pairs of isolates. A two-dimensional PCA plot was generated considering the first two highest eigenvalues, and genetic clusters were determined based on the eigenvector coordinates along the axes of variation.

## Supporting Information

S1 Fig Plots of delta *K* (*ΔK*) based on Evanno’s method for the determination of hypothetical ancestral population cluster (*K*) from the STRUCTURE analysis extracted using the STRUCTURE Harvester. (A) All STRUCTURE analyses of *P*. *knowlesi* infections strongly indicate the existence of two probable cluster subpopulations when analyses were performed with (A) 44 macaque and 167 human infections from Kapit, Sarawak (*K* = 2, *ΔK* = 936.75), (B) only 512 human infections from all 10 sampling sites (*K* = 2, *ΔK* = 174.94) and (C) all 44 macaque and 512 human infections from Malaysia (*K* = 2, *ΔK* = 136.39). (D) In a similar analysis, reanalysis of 404 macaque and human isolates from the Cluster 1 population did not resolve any further population clusters, as indicated by the very low values of delta *K*.
(DOCX)

S2 Fig Isolation-by-distance model of *P*. *knowlesi* from human infections of Cluster 1 and Cluster 2 subpopulations. Positive relationship between transformed genetic differentiation and natural log of geographical distance was observed for Cluster 1 subpopulation, which is represented by open circles (Mantel’s test of matrix correlation, P = 0.0016). In contrary, no significant relationship was observed for isolates from Cluster 2 subpopulation, which is represented in closed circles (Mantel’s test of matrix correlation, P = 0.0922). Due to limited number of samples in Cluster 2 subpopulation, isolates from each site of Sabah and Peninsular Malaysia were grouped together (total n = 9 and n = 5, respectively) to obtain the maximum number of samples prior to perform the test.
(DOCX)

S3 Fig Population genetic structure of *P*. *knowlesi* infections from all 512 humans, 34 long-tailed macaques and 10 pig-tailed macaques in Malaysia. (A) Bayesian model-based STRUCTURE analysis corresponds to those mention in Fig 3A where two subpopulation clusters were observed throughout the whole dataset (*K* = 2, *ΔK* = 136.39). When all of the human and macaque samples were analysed together, little evidence of admixture seen between parasites sampled in the two macaque populations. (B) The principal component analysis (PCA) of all *P*. *knowlesi* isolates also indicates the infections from different macaque host species were almost completely separated by the first principal component while human infections were widely distributed throughout the full range on both axes.
(DOCX)

S4 Fig Allele frequency distributions and genetic differentiations of 10 microsatellite loci between two *P*. *knowlesi* subpopulation clusters. Major subpopulation cluster (Cluster 1, n = 404) is colour-coded in black while minor subpopulation cluster (Cluster 2, n = 152) is in red.
(DOCX)

S5 Fig Re-running of Bayesian-approach STRUCTURE analysis on *P*. *knowlesi* of 512 human and 44 macaque infections using all microsatellite loci except for locus NC03_2 and locus CD13_61, which *F*_*ST*_ values > 0.3 between subpopulation clusters obtained from the initial STRUCTURE analysis results. (A) STRUCTURE analysis run with all loci except for locus NC03_2 indicated two subpopulation clusters throughout the whole dataset the (*K* = 2, *ΔK* = 142.50). (B) STRUCTURE analysis run with all loci except for locus CD13_61 indicated two subpopulation clusters throughout the whole dataset the (*K* = 2, *ΔK* = 23.17).
(DOCX)

S6 Fig Distribution of numbers of identical alleles out of 10 loci in pairwise comparisons of infections with intermediate cluster assignment indices above 0.25 from the overall STRUCTURE analysis. (DOCX)

S7 Fig Map illustrates the macaque sampling sites from locations within a 30 km radius of Kapit town, Sarawak. A total of 47 wild macaques were obtained within 30 km radius of Kapit town, Sarawak. Out of these, 37 were long-tailed macaques obtained near Kapit Waterboard from May 2004 to 2006 (n = 8), Rumah Braoh from March 2005 to January 2008 (n = 18), Sungai Seranau on July 2007 (n = 1), Sungai Mujong on August 2007 (n = 1), Sungai Sebabi on November 2007 (n = 1) Jalan Sungai Sut on March 2008 (n = 2), Rumah Belikau on March 2008 (n = 4) and Sungai Antaroh on March and April 2008 (n = 2). Ten pig-tailed macaques were obtained at Sungai Sut on March 2006 (n = 1), Rumah Untang near Sungai Yong from March to April 2008 (n = 8) and Sungai Setapang on April 2008 (n = 1). Map was accessed and modified from http://landsatlook.usgs.gov/.
(DOCX)

S1 Table Primers for genotyping of *P*. *knowlesi* microsatellites and location of loci in the reference genome sequence. (DOCX)

S2 Table Species-specificity of primers for 19 *P*. *knowlesi* microsatellite loci. (DOCX)

S3 Table Numbers of *P*. *knowlesi* infections genotyped and allelic diversity in each population. (DOCX)

S4 Table Genotypic data of 10 pairs and one triplet of identical haplotypes detected in six geographical locations. (DOCX)

S5 Table Degree of local Cluster 1 and Cluster 2 mixedness and test of multi-locus disequilibrium of *P*. *knowlesi* in human infections sampled at 10 sites across Malaysia. (DOCX)

S6 Table Pairwise measures of fixation indices (*F*_*ST*_ values above diagonal) and geographical distance (in kilometres below diagonal) across 10 populations of *P*. *knowlesi* from human infections. (DOCX)

S1 Dataset Genotypes at *P*. *knowlesi* microsatellite loci in infections from macaques (n = 47) and humans (n = 552). (XLSX)
