# Supplementary material for: Admixture in Humans of Two Divergent Plasmodium knowlesi Populations Associated with Different Macaque Host Species
Source: PLoS Pathog. 2015 May 28;11(5):e1004888. doi: 10.1371/journal.ppat.1004888 (PMC4447398; doi:10.1371/journal.ppat.1004888)
Supplement: S1 Fig — (A) All STRUCTURE analyses of P. knowlesi infections strongly indicate the existence of two probable cluster subpopulations when analyses were performed with (A) 44 macaque and 167 human infections from Kapit, Sarawak (K = 2, ΔK = 936.75), (B) only 512 human infections from all 10 sampling sites (K = 2, ΔK = 174.94) and (C) all 44 macaque and 512 human infections from Malaysia (K = 2, ΔK = 136.39). (D) In a similar analysis, reanalysis of 404 macaque and human isolates from the Cluster 1 population did not resolve any further population clusters, as indicated by the very low values of delta K. (DOCX) [file ppat.1004888.s001.docx]

**Figure S1.** Plots of delta *K* (*∆K*) based on Evanno’s method for the determination of hypothetical ancestral population cluster (*K*) from the STRUCTURE analysis extracted using the STRUCTURE Harvester. (A) All STRUCTURE analyses of *P. knowlesi* infections strongly indicate the existence of two probable cluster subpopulations when analyses were performed with (A) 44 macaque and 167 human infections from Kapit, Sarawak (*K* = 2, *∆K* = 936.75), (B) only 512 human infections from all 10 sampling sites (*K* = 2, *∆K* = 174.94) and (C) all 44 macaque and 512 human infections from Malaysia (*K* = 2, *∆K* = 136.39). (D) In a similar analysis, reanalysis of 404 macaque and human isolates from the Cluster 1 population did not resolve any further population clusters, as indicated by the very low values of delta *K*.

**A**


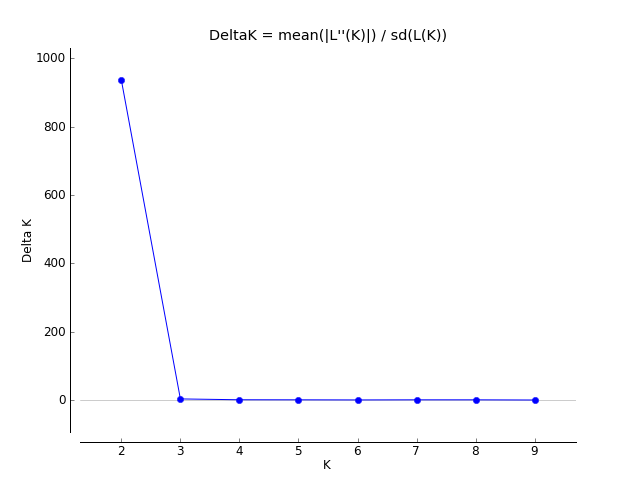


**B**


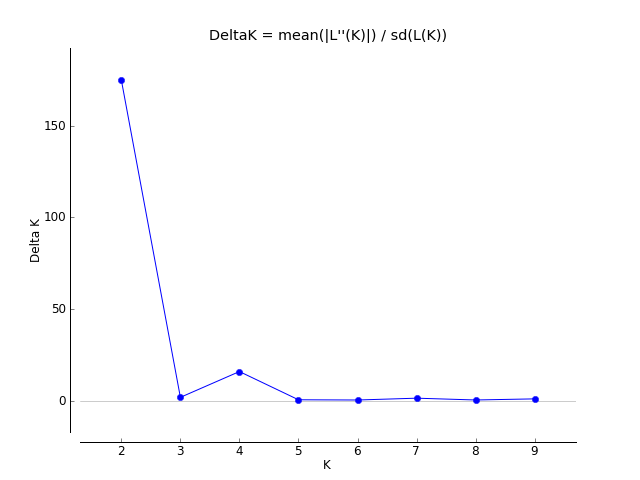


**C**

**
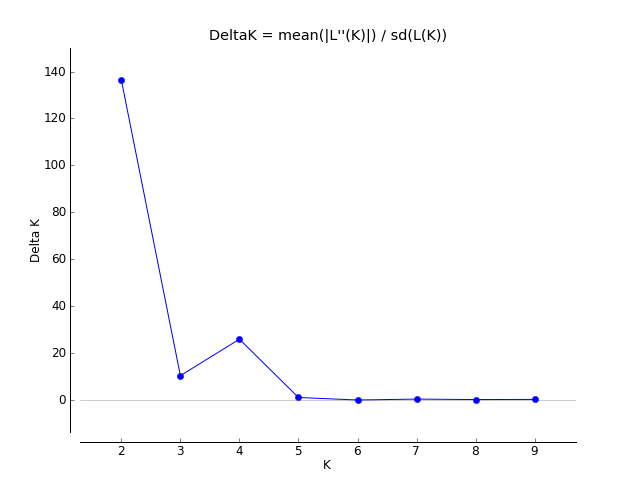
**

**D**

| **# K** | **Mean LnP(K)** | **Stdev LnP(K)** | **Ln'(K)** | **\|Ln''(K)\|** | **Delta K** |
| --- | --- | --- | --- | --- | --- |
| 1 | -517.95 | 0.55 | NA | NA | NA |
| 2 | -519.01 | 1.78 | -1.06 | 2.66 | 1.49 |
| 3 | -522.73 | 5.79 | -3.72 | 0.78 | 0.13 |
| 4 | -525.67 | 6.47 | -2.94 | 3.99 | 0.62 |
| 5 | -524.62 | 8.84 | 1.05 | 0.92 | 0.10 |
| 6 | -524.49 | 9.52 | 0.13 | 0.04 | 0.01 |
| 7 | -524.40 | 5.61 | 0.09 | 3.77 | 0.67 |
| 8 | -520.54 | 3.17 | 3.86 | 4.39 | 1.39 |
| 9 | -521.07 | 4.75 | -0.53 | 4.91 | 1.03 |
| 10 | -526.51 | 7.71 | -5.44 | 9.79 | 1.27 |
