# Supplementary material for: Admixture in Humans of Two Divergent Plasmodium knowlesi Populations Associated with Different Macaque Host Species
Source: PLoS Pathog. 2015 May 28;11(5):e1004888. doi: 10.1371/journal.ppat.1004888 (PMC4447398; doi:10.1371/journal.ppat.1004888)
Supplement: S2 Fig — Positive relationship between transformed genetic differentiation and natural log of geographical distance was observed for Cluster 1 subpopulation, which is represented by open circles (Mantel’s test of matrix correlation, P = 0.0016). In contrary, no significant relationship was observed for isolates from Cluster 2 subpopulation, which is represented in closed circles (Mantel’s test of matrix correlation, P = 0.0922). Due to limited number of samples in Cluster 2 subpopulation, isolates from each site of Sabah and Peninsular Malaysia were grouped together (total n = 9 and n = 5, respectively) to obtain the maximum number of samples prior to perform the test. (DOCX) [file ppat.1004888.s002.docx]

**Figure S2:** Isolation-by-distance model of *P. knowlesi* from human infections of Cluster 1 and Cluster 2 subpopulations.


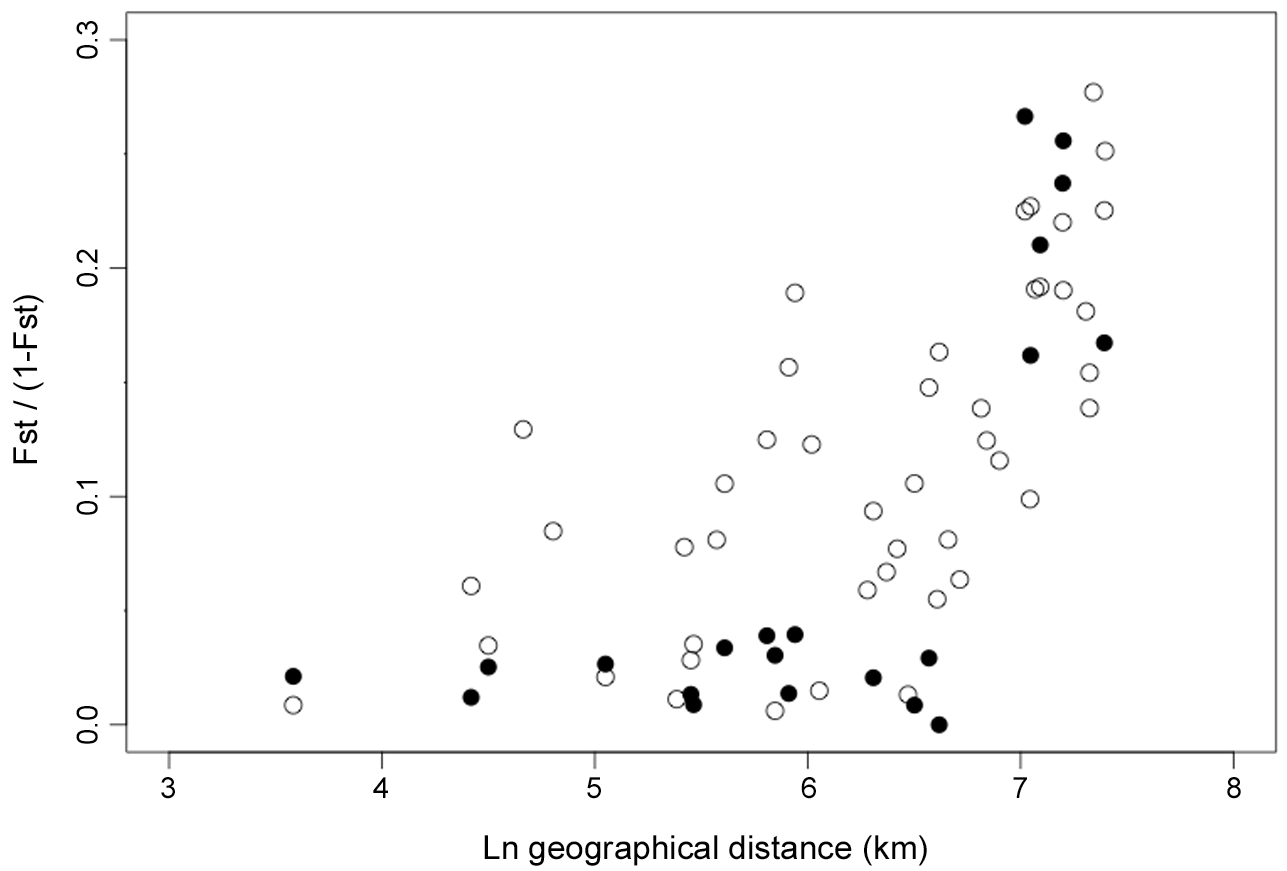


Positive relationship between transformed genetic differentiation and natural log of geographical distance was observed for Cluster 1 subpopulation, which is represented by open circles (Mantel’s test of matrix correlation, P = 0.0016). In contrary, no significant relationship was observed for isolates from Cluster 2 subpopulation, which is represented in closed circles (Mantel’s test of matrix correlation, P = 0.0922). Due to limited number of samples in Cluster 2 subpopulation, isolates from each site of Sabah and Peninsular Malaysia were grouped together (total n = 9 and n = 5, respectively) to obtain the maximum number of samples prior to perform the test.
