# Supplementary material for: Admixture in Humans of Two Divergent Plasmodium knowlesi Populations Associated with Different Macaque Host Species
Source: PLoS Pathog. 2015 May 28;11(5):e1004888. doi: 10.1371/journal.ppat.1004888 (PMC4447398; doi:10.1371/journal.ppat.1004888)

**Figure S3.** Population genetic structure of *P. knowlesi* infections from all 512 humans, 34 long-tailed macaques and 10 pig-tailed macaques in Malaysia. (A) Bayesian model-based STRUCTURE analysis corresponds to those mention in Figure 3A where two subpopulation clusters were observed throughout the whole dataset (*K* = 2, *∆K* = 136.39). When all of the human and macaque samples were analysed together, little evidence of admixture seen between parasites sampled in the two macaque populations. (B) The principal component analysis (PCA) of all *P. knowlesi* isolates also indicates the infections from different macaque host species were almost completely separated by the first principal component while human infections were widely distributed throughout the full range on both axes.


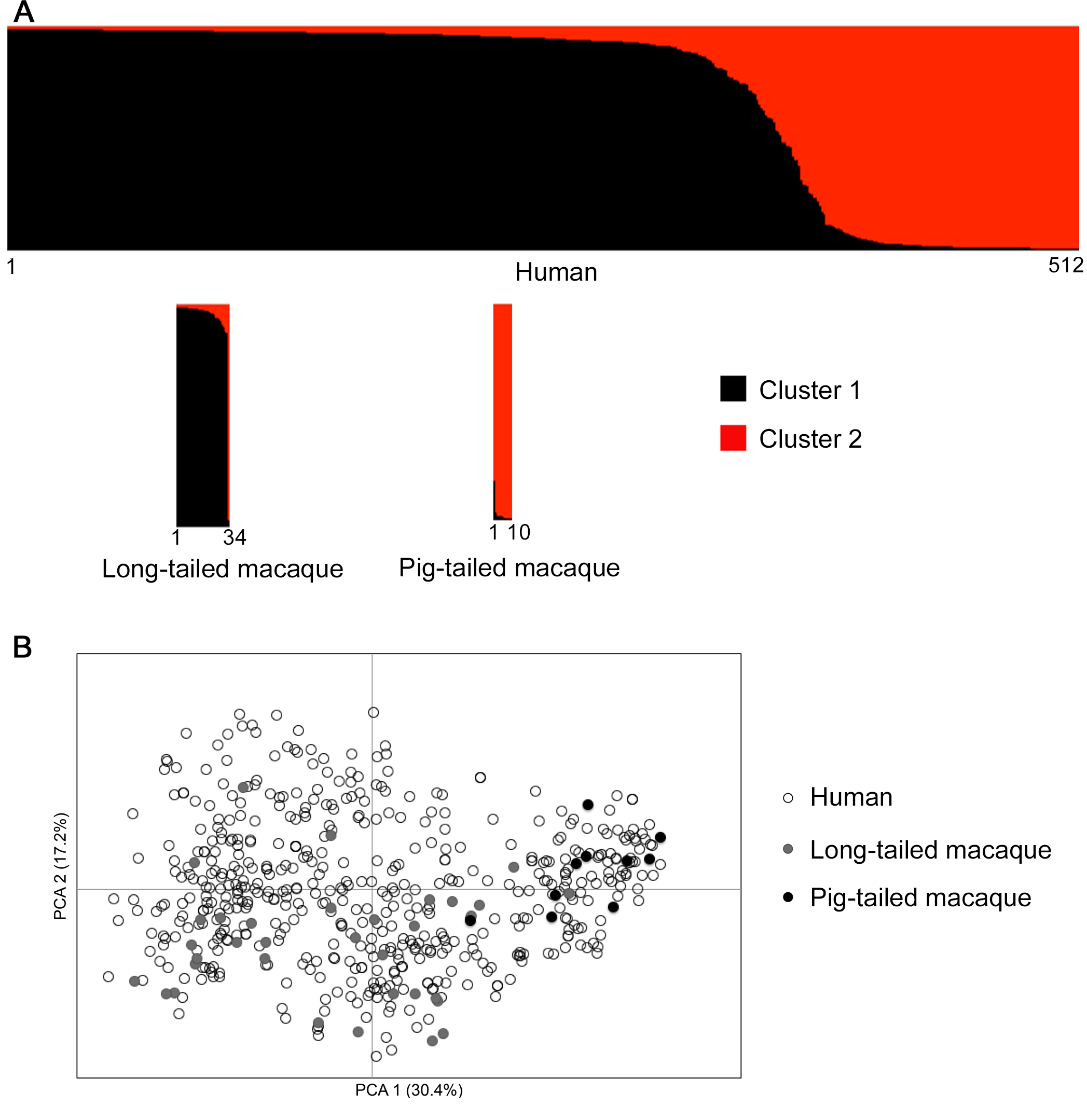

Supplement: S3 Fig — (A) Bayesian model-based STRUCTURE analysis corresponds to those mention in Fig 3A where two subpopulation clusters were observed throughout the whole dataset (K = 2, ΔK = 136.39). When all of the human and macaque samples were analysed together, little evidence of admixture seen between parasites sampled in the two macaque populations. (B) The principal component analysis (PCA) of all P. knowlesi isolates also indicates the infections from different macaque host species were almost completely separated by the first principal component while human infections were widely distributed throughout the full range on both axes. (DOCX) [file ppat.1004888.s003.docx]
