# Supplementary material for: Admixture in Humans of Two Divergent Plasmodium knowlesi Populations Associated with Different Macaque Host Species
Source: PLoS Pathog. 2015 May 28;11(5):e1004888. doi: 10.1371/journal.ppat.1004888 (PMC4447398; doi:10.1371/journal.ppat.1004888)
Supplement: S4 Fig — Major subpopulation cluster (Cluster 1, n = 404) is colour-coded in black while minor subpopulation cluster (Cluster 2, n = 152) is in red. (DOCX) [file ppat.1004888.s004.docx]

**Figure S4:** Allele frequency distributions and genetic differentiations of 10 microsatellite loci between two *P. knowlesi* subpopulation clusters.

| **Frequency** |  |  |
| --- | --- | --- |
|  |  |  |
|  |  |  |
|  |  |  |
|  |  |  |
|  | **Allele length (bp)** | |

Major subpopulation cluster (Cluster 1, n = 404) is colour-coded in black while minor subpopulation cluster (Cluster 2, n = 152) is in red.
