# Supplementary material for: Admixture in Humans of Two Divergent Plasmodium knowlesi Populations Associated with Different Macaque Host Species
Source: PLoS Pathog. 2015 May 28;11(5):e1004888. doi: 10.1371/journal.ppat.1004888 (PMC4447398; doi:10.1371/journal.ppat.1004888)
Supplement: S5 Fig — (A) STRUCTURE analysis run with all loci except for locus NC03_2 indicated two subpopulation clusters throughout the whole dataset the (K = 2, ΔK = 142.50). (B) STRUCTURE analysis run with all loci except for locus CD13_61 indicated two subpopulation clusters throughout the whole dataset the (K = 2, ΔK = 23.17). (DOCX) [file ppat.1004888.s005.docx]

**Figure S5:** Re-running of Bayesian-approach STRUCTURE analysis on *P. knowlesi* of 512 human and 44 macaque infections using all microsatellite loci except for locus NC03_2 and locus CD13_61, which *F_ST_* values > 0.3 between subpopulation clusters obtained from the initial STRUCTURE analysis results. (A) STRUCTURE analysis run with all loci except for locus NC03_2 indicated two subpopulation clusters throughout the whole dataset the (*K* = 2, *∆K* = 142.50). (B) STRUCTURE analysis run with all loci except for locus CD13_61 indicated two subpopulation clusters throughout the whole dataset the (*K* = 2, *∆K* = 23.17).

**A**

**B**
