# Supplementary material for: Admixture in Humans of Two Divergent Plasmodium knowlesi Populations Associated with Different Macaque Host Species
Source: PLoS Pathog. 2015 May 28;11(5):e1004888. doi: 10.1371/journal.ppat.1004888 (PMC4447398; doi:10.1371/journal.ppat.1004888)

**Figure S6.** Distribution of numbers of identical alleles out of 10 loci in pairwise comparisons of infections with intermediate cluster assignment indices above 0.25 from the overall STRUCTURE analysis.


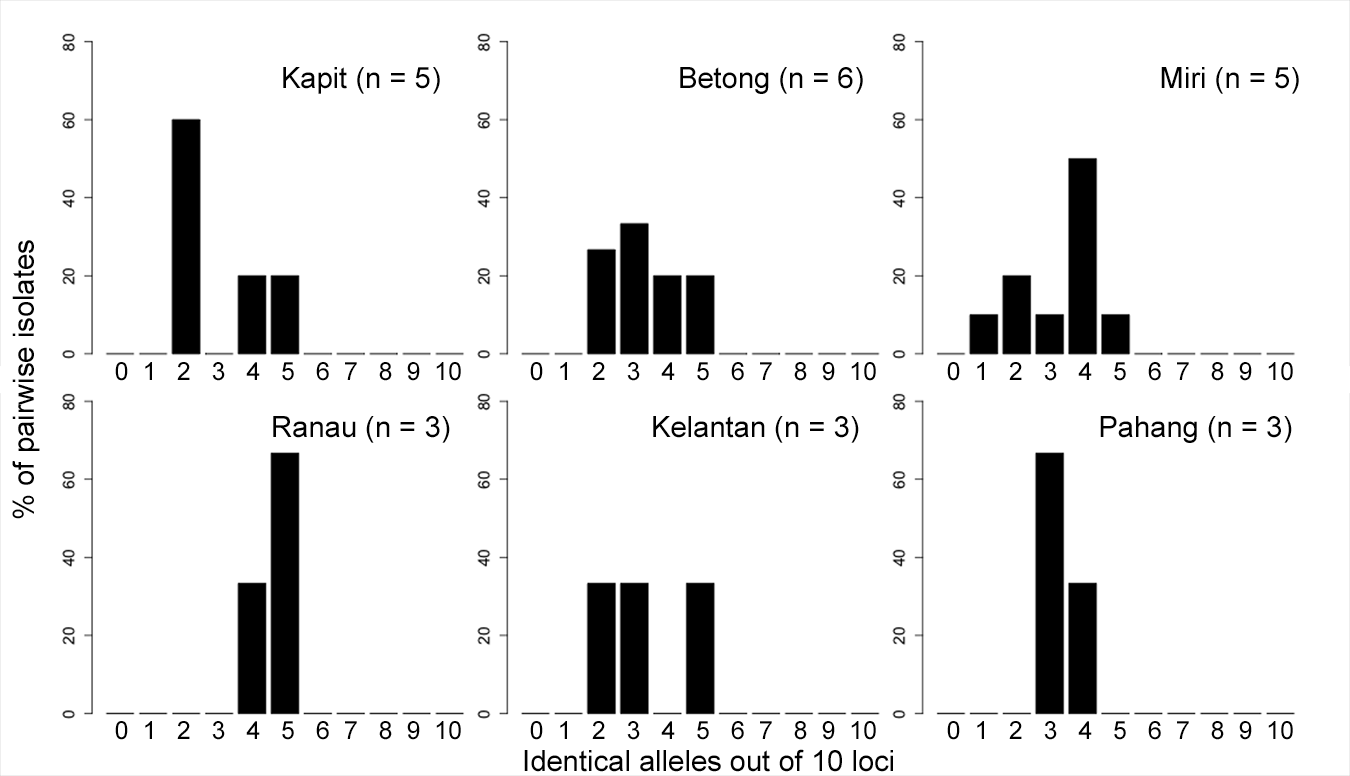

Supplement: S6 Fig — (DOCX) [file ppat.1004888.s006.docx]
