# Supplementary material for: Admixture in Humans of Two Divergent Plasmodium knowlesi Populations Associated with Different Macaque Host Species
Source: PLoS Pathog. 2015 May 28;11(5):e1004888. doi: 10.1371/journal.ppat.1004888 (PMC4447398; doi:10.1371/journal.ppat.1004888)
Supplement: S7 Fig — A total of 47 wild macaques were obtained within 30 km radius of Kapit town, Sarawak. Out of these, 37 were long-tailed macaques obtained near Kapit Waterboard from May 2004 to 2006 (n = 8), Rumah Braoh from March 2005 to January 2008 (n = 18), Sungai Seranau on July 2007 (n = 1), Sungai Mujong on August 2007 (n = 1), Sungai Sebabi on November 2007 (n = 1) Jalan Sungai Sut on March 2008 (n = 2), Rumah Belikau on March 2008 (n = 4) and Sungai Antaroh on March and April 2008 (n = 2). Ten pig-tailed macaques were obtained at Sungai Sut on March 2006 (n = 1), Rumah Untang near Sungai Yong from March to April 2008 (n = 8) and Sungai Setapang on April 2008 (n = 1). Map was accessed and modified from http://landsatlook.usgs.gov/. (DOCX) [file ppat.1004888.s007.docx]

**Figure S7.** Map illustrates the macaque sampling sites from locations within a 30 km radius of Kapit town, Sarawak.


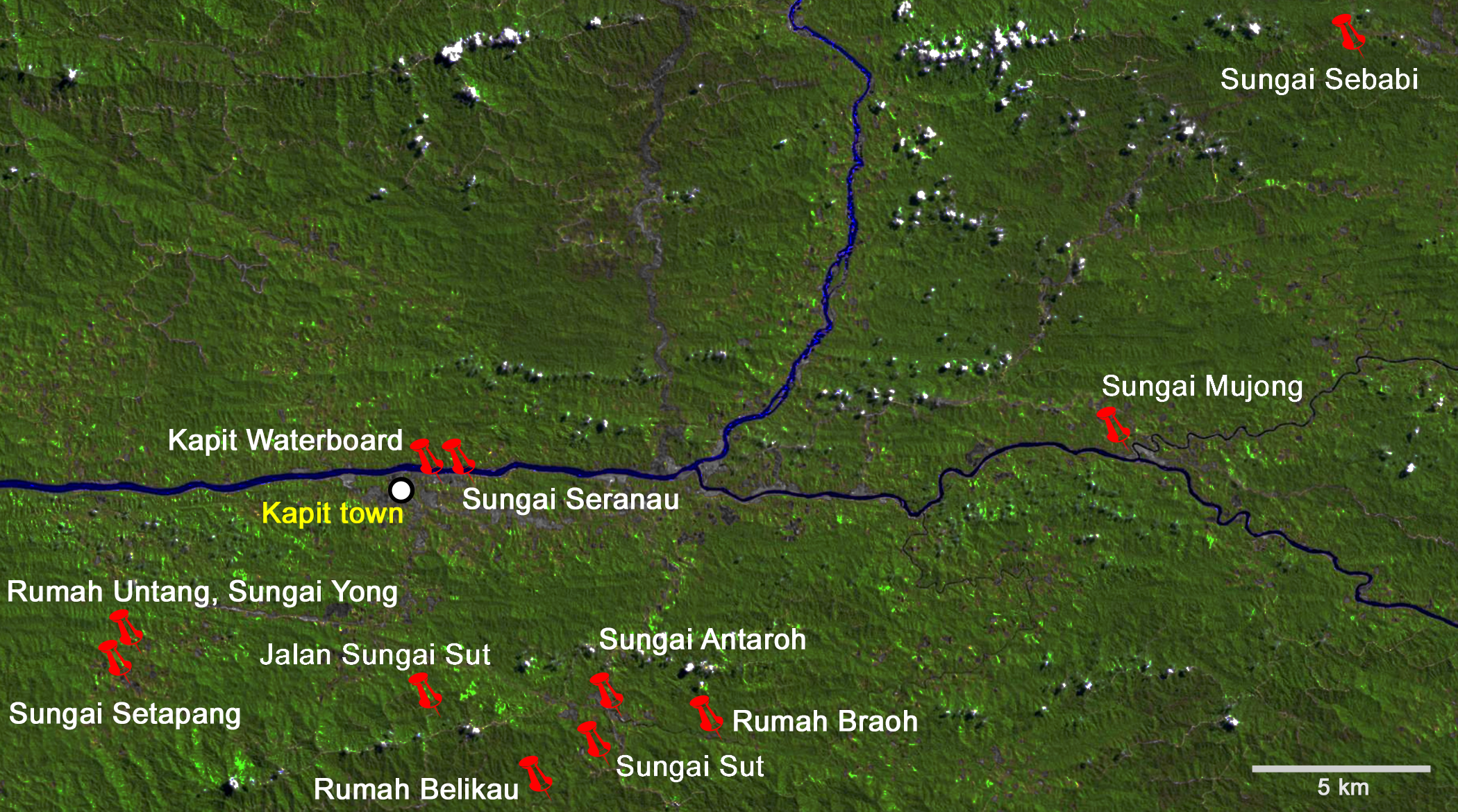


A total of 47 wild macaques were obtained within 30 km radius of Kapit town, Sarawak. Out of these, 37 were long-tailed macaques obtained near Kapit Waterboard from May 2004 to 2006 (n = 8), Rumah Braoh from March 2005 to January 2008 (n = 18), Sungai Seranau on July 2007 (n = 1), Sungai Mujong on August 2007 (n = 1), Sungai Sebabi on November 2007 (n = 1) Jalan Sungai Sut on March 2008 (n = 2), Rumah Belikau on March 2008 (n = 4) and Sungai Antaroh on March and April 2008 (n = 2). Ten pig-tailed macaques were obtained at Sungai Sut on March 2006 (n = 1), Rumah Untang near Sungai Yong from March to April 2008 (n = 8) and Sungai Setapang on April 2008 (n = 1). Map was accessed and modified from http://landsatlook.usgs.gov/.
