# Supplementary material for: Admixture in Humans of Two Divergent Plasmodium knowlesi Populations Associated with Different Macaque Host Species
Source: PLoS Pathog. 2015 May 28;11(5):e1004888. doi: 10.1371/journal.ppat.1004888 (PMC4447398; doi:10.1371/journal.ppat.1004888)
Supplement: S1 Table — (DOCX) [file ppat.1004888.s008.docx]

**Table S1.** Primers for genotyping of *P. knowlesi* microsatellites and location of loci in the reference genome sequence.

For each locus, the first two primers are used in the Nest 1 reaction, while the second and third primers are used in the Nest 2 reaction. In each primer ID, the F and R labels denote forward and reverse primers, respectively. The third primer for each locus is internal to the other two and labelled with a specific fluorescent dye for genotyping on the capillary electrophoresis.

**A.** Ten loci that were *P. knowlesi*-species specific and used for subsequent genotyping experiments.

| **Locus** | **Chromosome**  **(location of locus)*** | **Primer ID** | **Fluorescent dye label** | **Sequence**  **(5’ 🡪 3’)** |
| --- | --- | --- | --- | --- |
| NC03_2 | 3  (762,413 – 762,566) | N03_2R5  N03_2mF1  N03_2mR1 | **6FAM** | AGACTCATGTGCGGCGTTCCTT  GCGGGGAGGACGATAAACCATA  CGTCAAATGAAGAGAGCATTGCTC |
| CD05_06 | 5  (110,541 – 110,790) | C5R2  C5F2  C5R1 | **VIC** | GCTACAATGTTTGGAATCAGAAGG  GCCCATTGCAGCTATGCAC  GTTTTCGCTCCATGTTCAGCC |
| CD08_61 | 8  (943,277 – 943,507) | C8R7  C8F3  C8R5 | **VIC** | CTTGAACGTGCGTTTACATTTCC  GATCAGTGGACTGGTATACACAGATA  CCATGTAGGATGTATATTTCTTCG |
| NC09_1 | 9  (217,751 – 218,039) | N09_1mR2  N09_1F2  N09_mR1 | **PET** | TTCTCCACTTGACTTAAGGATTAAGC  TTGAAGCGGAATAGGGAAGGAT  CACACAGGTACGTGCATACATATAAG |
| NC10_1 | 10  (760,484 – 760,758) | N10_1mR2  N10_1F1  N10_1R2 | **NED** | ATGTAGTAATGTTGGGGCTGTTGGTG  CATGGCTGGTATCCCCCTGTTC  GGAAGGAGAGGACTAGTGCTGAAAGAG |
| CD11_157 | 11  (2,266,650 – 2,266,897) | C11bR3  C11bF2  C11R2 | **NED** | AATACATTTCGGGCAATATTCTCG  TAGGGATCGTCAGAGGGAGG  GTAGCAAATATGCTCGTTCAGG |
| NC12_2 | 12  (930,911 – 931,257) | N12_2F4  N12_2mR1  N12_2mF1 | **VIC** | TTCGTTGTTCTGTTTTTCCTTTGTTACT  ATGGATTCACTCACCTTGTGGTG  GTGGAAAGGAGGCAAACACACAG |
| NC12_4 | 12  (1,577,676 – 1,577,910) | N12_4mF2  N12_4mR2  N12_4mF1 | **VIC** | GTCCTGATGAAATTGAATTTGTGC  AGGGGTCGTATCGTCTCGTACG  GGAGTTCTTCCGTTCCGAATG |
| CD13_61 | 13  (774,903 – 775,077) | C13aF2  C13aR1  C13aF1 | **6FAM** | CCACTGATTGACAAGAAGAAGTTG  GTTCCAATTGTTGGCCCCATTG  CCAACAACCATTCAGTTGACAAAG |
| CD13_107 | 13  (1,220,220 – 1,220,405) | C13bR6  C13bF1  C13bR1 | **NED** | CGTGTGAAGAACGTAGTAGTACTG  GATGACCACGTTATGGATAATGTTG  CGTAAAACTCTGCACCTCCTTTGC |

*location of locus is based on primers used in second nest PCR

**B.** Nine *P. knowlesi* loci for which there was also some limited PCR amplification from DNA of one or more other species (Table S2), or stutter bands, precluding the breadth of application required in the current study.

| **Locus** | **Chromosome**  **(location of locus)*** | **Primer ID** | **Fluorescent dye label** | **Sequence**  **(5’ 🡪 3’)** |
| --- | --- | --- | --- | --- |
| NC02_2 | 2  (180,599 – 180,839) | N02_2F5  N02_2R1  N02_2F1 | **6FAM** | TGGCCAACAAGGGTAGCATCA  AATGCAGTTCTTTCCTTTACACGAG  AAGAGGCGAAGGAAGATAATCACATAGG |
| NC02_4 | 2  (76,039 – 76,381) | N02_4mR3  N02_4F1  N02_4mR2 |  | GGTATAACATTTTCATAACTCAGAAGAC  ACACTGTTACGACTTTTTCTTTCCATT  TGGTGTTGGAGGATCCATAGACA |
| CD03_40 | 3  (147,219 – 147,454) | C3F2  C3R1  C3F1 |  | ATAGAGCGTGAAAAGAACAAAGAG  ACCTCTCAATAGGGTAGGGTTTAG  CGATTCTGAAAAAAAGAAAGAAGAG |
| NC04_1 | 4  (60,354 – 60,595) | N04_1R7  N04_1mF2  N04_1R4 |  | GCGCGGCACCACCTTTTAT  GATTCGTTATTCTCGTGCACACAAG  CCCCAATTTGATGTATAAGGTAGCAGAG |
| NC05_2 | 5  (885,398 – 885,603) | N05_2R1  N05_2mF2  N05_2mR1 |  | ATTATTACAACTCATGGATGGAAGATTT  TCCCCTCACCTTGGGAGAGTG  GCAGTCGTTACGCGCGTTACAT |
| NC08_3 | 8  (69,052 – 69,207) | N08_3mR1  N08_3mF1  N08_3R1 | **VIC** | GATGAAGAATTTGTAGAGGCCG  CTTCGGTAGAAGGAAAAAACTGTG  ACCATGATGTTTATTGTAGGGCTGAA |
| NC10_5 | 10  (1,126,914 – 1,127,165) | N10_5mF3  N10_5mR1  N10_5mF2 |  | TGACCAGCTAGCCAATCTGTCA  TGCATGCACACGGTACCAATTA  CGGTCTGCATTTGTCCCG |
| CD11_86 | 11  (1,339,944 – 1,340,119) | C11aF2  C11aR1  C11aF1 |  | TGGGTGATGAGGTCGTTGAATAGG  GGTTGACGAGCAGGGTAAAACTGAG  TATGCCTGCGGGAAGGGTGAG |
| NC12_7 | 12  (2,483,130 – 2,483,440) | N12_7R1  N12_7F2  N12_7mR1 |  | TTCCCCCTCGTGCGACTCTTCT  AGGTAAGAGCCACGCAAGAATAACAACT  GGCTTGGCAGCTTTACTTAAGTTCC |

*location of locus is based on primers used in second nest PCR
