# Supplementary material for: Admixture in Humans of Two Divergent Plasmodium knowlesi Populations Associated with Different Macaque Host Species
Source: PLoS Pathog. 2015 May 28;11(5):e1004888. doi: 10.1371/journal.ppat.1004888 (PMC4447398; doi:10.1371/journal.ppat.1004888)
Supplement: S2 Table — (DOCX) [file ppat.1004888.s009.docx]

**Table S2.** Species-specificity of primers for 19 microsatellite loci. The specificity of primers were initially visualised under agarose gel, and those with single band were further confirmed using the GeneMapper software. All primers for each locus did not cross-react to *P. falciparum, P. vivax, P. malariae, P. ovale*, human and macaque DNA.

| **Locus ID** | **Chromosome** | **Repeat motifs in reference genome** | **Visualisation of species-specificity** | | |
| --- | --- | --- | --- | --- | --- |
|  |  |  | **Agarose gel** | **GeneMapper** |  |
| NC02_2 | 2 | (TTA)9 | Pk | Pk, with 1 bp stutter peaks |  |
| NC02_4 | 2 | (ATA)7 | Pk, Pin, Pfi | Not done |  |
| NC03_2 | 3 | (AAG)11 | Pk | Pk |  |
| CD03_40 | 3 | (AAG)11 | Pk | Pk, Pct, Pfi |  |
| NC04_1 | 4 | (GAA)10 | Pk, Pct | Not done |  |
| NC05_2 | 5 | (ATT)9 | Pk, Pct | Not done |  |
| CD05_06 | 5 | (TAA)7 | Pk | Pk |  |
| NC08_3 | 8 | (TCA)7 | Pk | Pk, Pcy, Pfi, Pct |  |
| CD08_61 | 8 | (TAC)11 | Pk | Pk |  |
| NC09_1 | 9 | (GAA)9 | Pk | Pk |  |
| NC10_1 | 10 | (TTA)9 | Pk | Pk |  |
| NC10_5 | 10 | (AAT)7 | Pk, Pfrg | Not done |  |
| CD11_86 | 11 | (CAA)7 | Pk | Pk, Pcy, Pct, Pin |  |
| CD11_157 | 11 | (GAG)8 | Pk | Pk |  |
| NC12_2 | 12 | (AAT)16 | Pk | Pk |  |
| NC12_4 | 12 | (GAA)7 | Pk | Pk |  |
| NC12_7 | 12 | (TAT)7 | Pk, Pct | Not done |  |
| CD13_61 | 13 | (AAC)8 | Pk | Pk |  |
| CD13_107 | 13 | (AGG)7 | Pk | Pk |  |

Pk – *P. knowlesi*, Pct – *P. coatneyi*, Pfi – *P. fieldi*, Pcy – *P. cynomolgi*, P. in – *P. inui*, Pfrg – *P. fragile*
