# Supplementary material for: Admixture in Humans of Two Divergent Plasmodium knowlesi Populations Associated with Different Macaque Host Species
Source: PLoS Pathog. 2015 May 28;11(5):e1004888. doi: 10.1371/journal.ppat.1004888 (PMC4447398; doi:10.1371/journal.ppat.1004888)
Supplement: S3 Table — (DOCX) [file ppat.1004888.s010.docx]

­

| **Table S3.** Numbers of *P. knowlesi* infections genotyped and allelic diversity in each population.  **A.** Numbers of infections genotyped in 10 human and 2 macaque populations across Malaysia using 10 microsatellite loci. | | | | | | | | | | | | | | | | | |
| --- | --- | --- | --- | --- | --- | --- | --- | --- | --- | --- | --- | --- | --- | --- | --- | --- | --- |
|  |  |  |  |  |  |  |  |  |  |  |  |  |  |  |  |  |  |
| **Locus** | **Sarawak** | | | | | | |  | **Sabah** | | |  | **Peninsular** | |  | **Total** | |
|  | **Long-tailed macaque** | **Pig-tailed macaque** | **Human** | **Human** | **Human** | **Human** | **Human** |  | **Human** | **Human** | **Human** |  | **Human** | **Human** |  |  |  |
|  | **Kapit** | **Kapit** | **Kapit** | **Betong** | **Kanowit** | **Sarikei** | **Miri** |  | **Kudat** | **Ranau** | **Tenom** |  | **Kelantan** | **Pahang** |  |  |  |
|  | **(n=37)** | **(n=10)** | **(n=185)** | **(n=78)** | **(n=34)** | **(n=27)** | **(n=50)** |  | **(n=30)** | **(n=42)** | **(n=26)** |  | **(n=30)** | **(n=50)** |  | **(n=599)** | **%** |
| NC12_2 | 36 | 10 | 171 | 72 | 34 | 24 | 50 |  | 28 | 39 | 26 |  | 28 | 50 |  | 568 | 94.8 |
| NC03_2 | 36 | 10 | 184 | 77 | 34 | 25 | 50 |  | 30 | 39 | 26 |  | 28 | 50 |  | 589 | 98.3 |
| NC09_1 | 36 | 10 | 182 | 76 | 34 | 26 | 50 |  | 29 | 40 | 26 |  | 27 | 50 |  | 586 | 97.8 |
| NC12_4 | 36 | 10 | 183 | 76 | 34 | 26 | 50 |  | 30 | 41 | 26 |  | 29 | 50 |  | 591 | 98.7 |
| NC10_1 | 37 | 10 | 184 | 77 | 34 | 26 | 50 |  | 30 | 41 | 26 |  | 27 | 50 |  | 592 | 98.8 |
| CD08_61 | 36 | 10 | 184 | 76 | 34 | 27 | 50 |  | 30 | 42 | 26 |  | 29 | 50 |  | 594 | 99.2 |
| CD11_157 | 34 | 10 | 183 | 78 | 34 | 26 | 50 |  | 30 | 40 | 26 |  | 28 | 50 |  | 589 | 98.3 |
| CD13_61 | 36 | 10 | 181 | 77 | 33 | 26 | 50 |  | 30 | 40 | 26 |  | 28 | 50 |  | 587 | 98.0 |
| CD05_06 | 36 | 10 | 183 | 77 | 34 | 26 | 50 |  | 30 | 40 | 26 |  | 29 | 50 |  | 591 | 98.7 |
| CD13_107 | 36 | 10 | 183 | 77 | 34 | 26 | 50 |  | 30 | 39 | 26 |  | 27 | 50 |  | 588 | 98.2 |
| All loci | 34 | 10 | 167 | 71 | 33 | 24 | 50 |  | 28 | 38 | 26 |  | 25 | 50 |  | 556 | 92.8 |

| **B.** Allelic diversity, measured as expected heterozygosity (*H_E_*), of 10 microsatellite loci of *P. knowlesi* in 12 populations across Malaysia. The data was analysed based on the single clone samples and only predominant alleles in the multiple clone infections. | | | | | | | | | | | | | | |
| --- | --- | --- | --- | --- | --- | --- | --- | --- | --- | --- | --- | --- | --- | --- |
|  | | | | | |  |  |  |  |  |  |  |  |  |
| **Locus** | **Sarawak** | | | | | | |  | **Sabah** | | |  | **Peninsular** | |
|  | **Long-tailed macaque** | **Pig-tailed macaque** | **Human** | **Human** | **Human** | **Human** | **Human** |  | **Human** | **Human** | **Human** |  | **Human** | **Human** |
|  | **Kapit** | **Kapit** | **Kapit** | **Betong** | **Kanowit** | **Sarikei** | **Miri** |  | **Kudat** | **Ranau** | **Tenom** |  | **Kelantan** | **Pahang** |
|  | **(n = 37)** | **(n = 10)** | **(n = 185)** | **(n = 78)** | **(n = 34)** | **(n = 27)** | **(n = 50)** |  | **(n = 30)** | **(n = 42)** | **(n = 26)** |  | **(n = 30)** | **(n = 50)** |
| NC12_2 | 0.85 | 0.82 | 0.86 | 0.85 | 0.86 | 0.84 | 0.84 |  | 0.84 | 0.90 | 0.86 |  | 0.80 | 0.92 |
| NC03_2 | 0.57 | 0.00 | 0.51 | 0.55 | 0.43 | 0.63 | 0.51 |  | 0.54 | 0.63 | 0.58 |  | 0.48 | 0.60 |
| NC09_1 | 0.78 | 0.78 | 0.89 | 0.85 | 0.89 | 0.92 | 0.85 |  | 0.90 | 0.77 | 0.87 |  | 0.54 | 0.63 |
| NC12_4 | 0.86 | 0.51 | 0.84 | 0.85 | 0.74 | 0.80 | 0.77 |  | 0.81 | 0.81 | 0.77 |  | 0.63 | 0.70 |
| NC10_1 | 0.65 | 0.78 | 0.73 | 0.72 | 0.57 | 0.67 | 0.65 |  | 0.53 | 0.61 | 0.66 |  | 0.76 | 0.83 |
| CD08_61 | 0.82 | 0.00 | 0.67 | 0.15 | 0.47 | 0.14 | 0.42 |  | 0.83 | 0.82 | 0.85 |  | 0.83 | 0.80 |
| CD11_157 | 0.77 | 0.84 | 0.80 | 0.79 | 0.81 | 0.88 | 0.77 |  | 0.76 | 0.84 | 0.80 |  | 0.67 | 0.75 |
| CD13_61 | 0.52 | 0.36 | 0.76 | 0.73 | 0.82 | 0.72 | 0.66 |  | 0.69 | 0.73 | 0.67 |  | 0.77 | 0.72 |
| CD05_06 | 0.81 | 0.60 | 0.78 | 0.80 | 0.80 | 0.82 | 0.74 |  | 0.73 | 0.79 | 0.55 |  | 0.78 | 0.70 |
| CD13_107 | 0.47 | 0.76 | 0.63 | 0.41 | 0.82 | 0.72 | 0.76 |  | 0.13 | 0.43 | 0.50 |  | 0.73 | 0.56 |
| Mean *H_E_* | 0.71 | 0.54 | 0.75 | 0.67 | 0.72 | 0.71 | 0.70 |  | 0.67 | 0.73 | 0.71 |  | 0.70 | 0.72 |
| SE | 0.05 | 0.10 | 0.04 | 0.07 | 0.05 | 0.07 | 0.04 |  | 0.07 | 0.04 | 0.04 |  | 0.04 | 0.04 |
