# Supplementary material for: Admixture in Humans of Two Divergent Plasmodium knowlesi Populations Associated with Different Macaque Host Species
Source: PLoS Pathog. 2015 May 28;11(5):e1004888. doi: 10.1371/journal.ppat.1004888 (PMC4447398; doi:10.1371/journal.ppat.1004888)
Supplement: S4 Table — (DOCX) [file ppat.1004888.s011.docx]

**Table S4.** Genotypic data of 10 pairs and one triplet of identical haplotypes detected in six geographical locations. Out of 11 haplotypes, all were unique with no identical genotype at all complete 10 loci.

| **Haplotype** | **Sample ID** | **Origin** | **Date of collection** | **Locus (with allele length in bp)** | | | | | | | | | | **Inferred cluster** |
| --- | --- | --- | --- | --- | --- | --- | --- | --- | --- | --- | --- | --- | --- | --- |
|  |  |  |  | **NC12_2** | **NC03_2** | **NC09_1** | **NC12_4** | **NC10_1** | **CD08_61** | **CD11_157** | **CD13_61** | **CD05_06** | **CD13_107** |  |
| 1 | KT033 | Kapit | 2013 | 316 | 129 | 293 | 226 | 258 | 213 | 248 | 170 | 266 | 186 | 1 |
|  | KT034 |  | 2013 |  |  |  |  |  |  |  |  |  |  |  |
|  | KT052 |  | 2013 |  |  |  |  |  |  |  |  |  |  |  |
| 2 | CDK158 | Kapit | 2007 | 319 | 129 | 305 | 229 | 261 | 228 | 251 | 170 | 266 | 186 | 1 |
|  | CDK185 |  | 2007 |  |  |  |  |  |  |  |  |  |  |  |
| 3 | BTG095 | Betong | 2013 | 313 | 129 | 278 | 241 | 261 | 228 | 242 | 167 | 251 | 189 | 1 |
|  | BTG098 |  | 2013 |  |  |  |  |  |  |  |  |  |  |  |
| 4 | BTG090 | Betong | 2013 | 316 | 129 | 284 | 238 | 258 | 213 | 248 | 170 | 251 | 186 | 1 |
|  | BTG091 |  | 2013 |  |  |  |  |  |  |  |  |  |  |  |
| 5 | BTG059 | Betong | 2012 | 316 | 132 | 278 | 229 | 264 | 213 | 254 | 158 | 248 | 183 | 2 |
|  | BTG063 |  | 2012 |  |  |  |  |  |  |  |  |  |  |  |
| 6 | DFS612 | Sarikei | 2011 | 319 | 132 | 290 | 226 | 258 | 213 | 263 | 158 | 248 | 174 | 2 |
|  | DFS613 |  | 2011 |  |  |  |  |  |  |  |  |  |  |  |
| 7 | MRI/028/03 | Miri | 2003 | 316 | 129 | 278 | 232 | 264 | 213 | 248 | 158 | 248 | 189 | 2 |
|  | MRI/097/04 |  | 2004 |  |  |  |  |  |  |  |  |  |  |  |
| 8 | MRI/021/02 | Miri | /2002 | 322 | 132 | 287 | 226 | 258 | 213 | 248 | 167 | 248 | 189 | 2 |
|  | MRI/083/04 |  | 2004 |  |  |  |  |  |  |  |  |  |  |  |
| 9 | MRI/093/04 | Miri | 2004 | 334 | 129 | 287 | 232 | 258 | 213 | 248 | 158 | 248 | 180 | 2 |
|  | MRI/096/04 |  | 2004 |  |  |  |  |  |  |  |  |  |  |  |
| 10 | ML146/13 | Tenom | 2013 | 322 | 132 | 284 | 235 | 258 | 219 | 245 | 170 | 248 | 189 | 1 |
|  | ML148/13 |  | 2013 |  |  |  |  |  |  |  |  |  |  |  |
| 11 | KEL25 | Kelantan | 2013 | 310 | 132 | 281 | 229 | 261 | 213 | 248 | 182 | 248 | 174 | 2 |
|  | KEL26 |  | 2013 |  |  |  |  |  |  |  |  |  |  |  |
