# Supplementary material for: Admixture in Humans of Two Divergent Plasmodium knowlesi Populations Associated with Different Macaque Host Species
Source: PLoS Pathog. 2015 May 28;11(5):e1004888. doi: 10.1371/journal.ppat.1004888 (PMC4447398; doi:10.1371/journal.ppat.1004888)
Supplement: S5 Table — (DOCX) [file ppat.1004888.s012.docx]

**Table S5.** Degree of local Cluster 1 and Cluster 2 mixedness and test of multi-locus disequilibrium of *P. knowlesi* in human infections sampled at 10 sites across Malaysia.

**A.** Proportion of human *P. knowlesi* infections designated as Cluster 1 and Cluster 2 in 10 geographical sites.

| **Regions** | **Sites** | **Frequency of Cluster 1 (*p_1_*)** | **Frequency of Cluster 2 (*p_2_*)** | **Degree of cluster mixedness (*p_1_*p_2_*)** |
| --- | --- | --- | --- | --- |
| Sarawak | Kapit | 0.71 | 0.29 | 0.21 |
| Sarawak | Betong | 0.82 | 0.18 | 0.15 |
| Sarawak | Kanowit | 0.45 | 0.55 | 0.25 |
| Sarawak | Sarikei | 0.58 | 0.42 | 0.24 |
| Sarawak | Miri | 0.26 | 0.74 | 0.19 |
| Sabah | Kudat | 0.96 | 0.04 | 0.04 |
| Sabah | Ranau | 0.89 | 0.11 | 0.10 |
| Sabah | Tenom | 0.85 | 0.15 | 0.13 |
| Peninsular | Kelantan | 0.84 | 0.16 | 0.13 |
| Peninsular | Pahang | 0.98 | 0.02 | 0.02 |

Pearson’s *X^2^* for test of homogeneity of proportions across populations, P < 0.0001

**B.** Test of multi-locus linkage disequilibrium of *P. knowlesi* isolated in humans by measuring the standardised index of association (*I_A_^S^*) using only unique haplotypes in each geographical sites

| **Regions** | **Sites** | **n** | ***I_A_^S^*** | **P-value** |
| --- | --- | --- | --- | --- |
| Sarawak | Kapit | 164 | 0.024 | <0.001 |
| Sarawak | Betong | 68 | 0.012 | 0.035 |
| Sarawak | Kanowit | 33 | 0.024 | 0.005 |
| Sarawak | Sarikei | 23 | 0.016 | 0.090 |
| Sarawak | Miri | 47 | 0.027 | <0.001 |
| Sabah | Kudat | 28 | 0.012 | 0.133 |
| Sabah | Ranau | 38 | 0.019 | 0.008 |
| Sabah | Tenom | 25 | 0.011 | 0.185 |
| Peninsular | Kelantan | 24 | 0.013 | 0.167 |
| Peninsular | Pahang | 50 | -0.001 | 0.564 |
