# Supplementary material for: Admixture in Humans of Two Divergent Plasmodium knowlesi Populations Associated with Different Macaque Host Species
Source: PLoS Pathog. 2015 May 28;11(5):e1004888. doi: 10.1371/journal.ppat.1004888 (PMC4447398; doi:10.1371/journal.ppat.1004888)
Supplement: S6 Table — (DOCX) [file ppat.1004888.s013.docx]

**Table S6.** Pairwise measures of fixation indices (*F_ST_* values above diagonal) and geographical distance (in kilometres below diagonal) across 10 populations of *P. knowlesi* from human infections.

| **Regions** | **Hosts** | **Sampling sites** | **Sarawak** | | | | |  | **Sabah** | | |  | **Peninsular** | |
| --- | --- | --- | --- | --- | --- | --- | --- | --- | --- | --- | --- | --- | --- | --- |
|  |  |  | **Human** | **Human** | **Human** | **Human** | **Human** |  | **Human** | **Human** | **Human** |  | **Human** | **Human** |
|  |  |  | **Kapit** | **Betong** | **Kanowit** | **Sarikei** | **Miri** |  | **Kudat** | **Ranau** | **Tenom** |  | **Kelantan** | **Pahang** |
| Sarawak | Human | Kapit | - | 0.025** | 0.016* | 0.011 | 0.041** |  | 0.041** | 0.062** | 0.015 |  | 0.127** | 0.086** |
| Sarawak | Human | Betong | 233 | - | 0.053** | 0.014 | 0.084** |  | 0.065** | 0.107** | 0.053** |  | 0.155** | 0.102** |
| Sarawak | Human | Kanowit | 156 | 90 | - | 0.008 | 0.019* |  | 0.094** | 0.079** | 0.052** |  | 0.145** | 0.123** |
| Sarawak | Human | Sarikei | 236 | 36 | 83 | - | 0.025 |  | 0.089** | 0.084** | 0.045 |  | 0.153** | 0.114** |
| Sarawak | Human | Miri | 273 | 380 | 333 | 369 | - |  | 0.127** | 0.074** | 0.071** |  | 0.164** | 0.147** |
| Sabah | Human | Kudat | 646 | 824 | 741 | 780 | 411 |  | - | 0.102** | 0.019 |  | 0.186** | 0.119** |
| Sabah | Human | Ranau | 549 | 748 | 666 | 713 | 346 |  | 106 | - | 0.058* |  | 0.150** | 0.132** |
| Sabah | Human | Tenom | 426 | 614 | 534 | 584 | 226 |  | 218 | 122 | - |  | 0.174** | 0.117** |
| Peninsular | Human | Kelantan | 1340 | 1149 | 1202 | 1119 | 1336 |  | 1631 | 1625 | 1544 |  | - | 0.059** |
| Peninsular | Human | Pahang | 1146 | 935 | 993 | 911 | 1174 |  | 1516 | 1490 | 1516 |  | 263 | - |
| Asterisks indicate pairwise tests of significance of differences between geographical populations (against null hypothesis of *F_ST_* = 0). Each test was performed using 66,000 permutations of random resampling of a combined population. Double asterisks (**) indicate P < 0.0001); single asterisks (*) indicate P between 0.0001 and 0.0008. Indicative adjusted nominal level of significance (α = 0.05) for multiple comparisons was P = 0.0008 after Bonferroni correction. | | | | | | | | | | | | | | |
